# Isolation and Characterization of Human Colon Adenocarcinoma Stem-Like Cells Based on the Endogenous Expression of the Stem Markers

**DOI:** 10.3390/ijms22094682

**Published:** 2021-04-28

**Authors:** Sergei A. Koshkin, Olga V. Anatskaya, Alexander E. Vinogradov, Vladimir N. Uversky, Guy W. Dayhoff, Margarita A. Bystriakova, Valery A. Pospelov, Elena N. Tolkunova

**Affiliations:** 1 Institute of Cytology of the Russian Academy of Science, 194064 St-Petersburg, Russia; margarita.bystryakova@gmail.com (M.A.B.); Pospelov_v@mail.ru (V.A.P.); 2 Department of Medical Oncology, Sidney Kimmel Cancer Center, Thomas Jefferson University, 1015 Walnut Street, Ste. 1024, Philadelphia, PA 19107, USA; 3 Department of Molecular Medicine and USF Health Byrd Alzheimer’s Research Institute, Morsani College of Medicine, University of South Florida, Tampa, FL 33612, USA; 4 Department of Chemistry, College of Art and Sciences, University of South Florida, Tampa, FL 33620, USA; gdayhoff@usf.edu

**Keywords:** colon cancer, cancer stem-like cells, OCT4, SORE6xreporter, mRNA seq, intrinsic disorder predisposition analysis

## Abstract

Background: Cancer stem cells’ (CSCs) self-maintenance is regulated via the pluripotency pathways promoting the most aggressive tumor phenotype. This study aimed to use the activity of these pathways for the CSCs’ subpopulation enrichment and separating cells characterized by the OCT4 and SOX2 expression. Methods: To select and analyze CSCs, we used the SORE6x lentiviral reporter plasmid for viral transduction of colon adenocarcinoma cells. Additionally, we assessed cell chemoresistance, clonogenic, invasive and migratory activity and the data of mRNA-seq and intrinsic disorder predisposition protein analysis (IDPPA). Results: We obtained the line of CSC-like cells selected on the basis of the expression of the OCT4 and SOX2 stem cell factors. The enriched CSC-like subpopulation had increased chemoresistance as well as clonogenic and migration activities. The bioinformatic analysis of mRNA seq data identified the up-regulation of pluripotency, development, drug resistance and phototransduction pathways, and the downregulation of pathways related to proliferation, cell cycle, aging, and differentiation. IDPPA indicated that CSC-like cells are predisposed to increased intrinsic protein disorder. Conclusion: The use of the SORE6x reporter construct for CSCs enrichment allows us to obtain CSC-like population that can be used as a model to search for the new prognostic factors and potential therapeutic targets for colon cancer treatment.

## 1. Introduction

Colon cancer remains one of the most frequent cancers in the developed countries, following lung and prostate cancers among males, and breast cancer among females [1]. Despite the availability of a wide range of drugs, the disease at the IV metastatic stage remains incurable, and the five-year survival of these patients does not exceed 5–10% [2,3]. Therefore, the study of malignant tumors in order to find new therapeutic targets remains one of the priorities of the modern medical and biological community.

Considerable attention is focused on study of the mechanisms of occurrence and maintenance of the intra-tumor heterogeneity [4]. According to the concept of cancer stem cells (CSCs), the growth and metastasis of the tumor are caused by the functioning of a small population of tumor cells with specific properties. These cells should have a number of functional characteristics, namely, a high ability to form tumors in the body of immunosuppressed mice when administered in limited quantities, the ability of self-maintenance and proliferation, and increased resistance to cytostatics. There are some similarities and differences between CSCs and healthy stem cells. The exclusive ability of CSCs to self-renew and to form the heterogeneous lineages of cancer cells that comprise the tumor bears resemblance to normal stem cells (NSCs). Targeting CSCs is an appealing approach for treating cancer [5,6,7].

With the isolation and characterization of CSCs from different solid tumors, CSCs are regarded now as a subset of the cells that have the ability of self-renewal and generation of heterogeneous tumor cells. However, mechanisms underlying the CSCs self-renewal regulation remain mostly unknown [8]. While pluripotency in regulating genes of NSCs (such as *Oct4* and *Nanog*) was shown to regulate the CSCs self-renewal, noticeable differences exist between the NSCs and CSCs in their self-renewal regulation [9,10,11]. For colon cancer, the most actively studied markers of the cancer stem cell are: CD133+ [12,13], CD44+ [14,15], EpCAM [16], CD29, CD24 [17], CD166 [18], ALDH1A1+ [19], and ALDH1B1 [20]. The simultaneous use of several markers for “stem cell-like” subpopulation enrichment allows isolation of the stem subpopulation of tumor cells more reliably and accurately [21].

The methods of enrichment based on the separation of cells with a pronounced manifestation of one of the CSCs’ functional properties are also described. An example is the isolation of cells capable of actively ejecting Hoechst dye via ATP-dependent ABC transporters, by fluorescence activated cell sorting (FACS), or antibodies conjugated to magnetic beads (MACS) [22], or isolation of radioresistant cells [23]. The stem component enrichment method based on the expression of aldehyde dehydrogenase (ALDH), for example, using the ALDEFLUOR set [24], is also reported. Despite the simplicity of these methods, not all obtained cells have a full set of CSC properties. For example, it is not always possible for them to form tumors in immune-deficient mice [25]. It is assumed, in particular, that proteolytic enzymes used in the process of cell isolation from tumor tissue cause damage to a significant part of the surface receptors [26]. The most complex method, but the one increasingly gaining popularity, is an enrichment based on the identification of cells expressing embryonic transcription factors. As a rule, this is implemented through the use of reporters containing parts of the promoter and enhancer regions of embryonic transcription factor [27]. In our work, we used the SORE6x genetic construct developed by the group of Binwu Tang [28]. This reporter contains six times repeated sequence of the *Nanog* gene promoter which is a binding site for the OCT4 and SOX2 key transcription factor of pluripotency. The use of a destabilized version of the GFP-dsCapGFP protein, which has a short half-life in the cell, can increase the sensitivity of reporter cells. Previously, the use of this reporter construct for enrichment of CSCs of various tumors was described. The great advantage of the reporter constructs is the ability to indirectly monitor the promoter activity in living cells in vitro or in vivo, during various functional experiments studying the mechanisms of the CSCs’ formation and searching for the factors that stimulate their formation [29]. There is growing evidence that the CSC state is dynamic and amenable to rapid transitions [29,30]. In a number of papers, the appearance of the stem phenotype in differentiated cells (phenotype change) in response to the various pathological and physiological effects was noted [31]. A body of studies revealed the promoting effects of the epithelial-mesenchymal transition (EMT) on the stemness of CSCs [32,33,34,35]. Similar to the embryonic development, EMT plays a role in wound healing and development of some diseases, such as fibrosis. In cancers, EMT is both associated with tumor metastasis, and also with the acquisition of tumor cells with stem cell properties [36,37].

Clarification of the genetic, epigenetic, and external conditions that allow the implementation of broad phenotypic plasticity contributing to the activation of the mechanisms of self-maintenance and protection of embryonic stem cells will significantly advance the treatment of common forms of malignant tumors.

## 2. Results

### 2.1. Enrichment of Cancer Stem-Like Cells from the Primary Culture of the Human Colon Cancer

**Figure 1:**
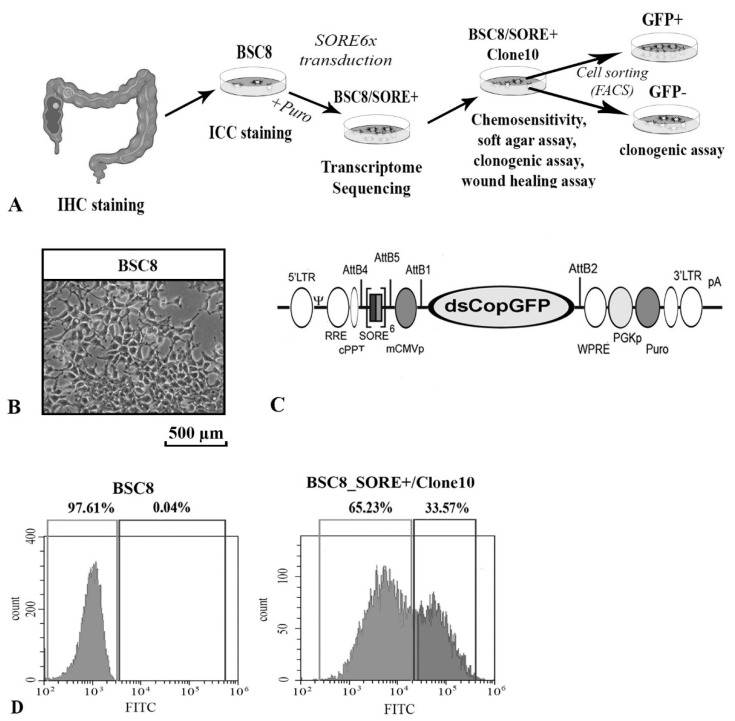
Obtaining a heterogeneous population of tumor stem cells. (**A**) Schematic representation of the experiment design. The source of the primary BSC8 cell line was human colon adenocarcinoma. CSC-like cells were obtained by viral transduction of the SORE6x reporter construct and subsequent selection on puromycin. (**B**). The BSC8 line cells in the culture. A polygonal cell shape typical to epithelial cells is noted. (**C**). SORE6x reporter plasmid constructed. The dsCopGFP gene and the Puro gene are under the control of the six-fold repeated responsive element from the Nanog gene promoter. (**D**). Histogram of the BSC8 and BSC8_SORE+/Clone 10 cell distribution according to the degree of fluorescence. The presence of two populations of the BSC8_SORE+/Clone 10 cells is determined, and the levels of fluorescence of these populations differed 100 times.

The use of primary cell culture allowed us to reduce the influence of culture factors on the biological characteristics of tumor cells. The selection of material for work, verification of the histological diagnosis, and interpretations of the immunohistochemistry (IHC) staining results were conducted by a certified pathologist. The histological structure of the tumor serving as a source of the cells corresponded to a moderately differentiated adenocarcinoma (Figure 1A,B, Supplementary Figure S1). Additionally, it was found that more than 35% of the cells expressed the ALDH1A1 stem cell marker.

To enrich a subpopulation of stem-like cells, we used the method proposed by Tang in 2015 [28] based on the SORE6x reporter construct introduction into the cells. The SORE6x plasmid contains a six-time repeated region of the *Nanog* gene promoter, which is a binding site for the endogenously expressed OCT4 and SOX2 transcription factors (Figure 1C). In the original paper, the authors also pointed out that the design allows “discarding” the products of the pseudogenes of the *Oct4* gene, which makes its use a highly effective method for the CSC enrichment. Activation of the reporter leads to the puromycin resistance and the expression of the destabilized form of GFP (with four-times shorter half-life than the original GFP). The use of these properties allowed us to select subpopulation of puromycin-resistant cells and analyze their CSCs-like properties comparing them with the cells of the original BSC8 line. After viral transduction and selection on the medium with puromycin during 5 days, more than 98% of cells died. The remaining puromycin-resistant cancer cells were called BSC8_SORE+and used for the transcriptome analysis.

We assumed that the resulting subpopulation of cells has properties similar to stem cells, based on the fact that the reporter structure assumed the acquisition of resistance only by cells with endogenous expression of recognized stem factors OCT4 and SOX2. It is known that cancer stem cells, according to their definition, are responsible for the formation of tumors and maintaining their growth, and one of their properties is increased resistance to cytostatics used in cancer therapy. Based on the property of increased resistance to 5-fluorouracil compared to the original cell line, we performed subcloning of the resulting population of puromycin-resistant cells and compared the chemoresistance of the obtained subclones.

### 2.2. The Increased Cytostatic Resistance of Cell Clones Selected after Reporter Transduction

**Figure 2:**
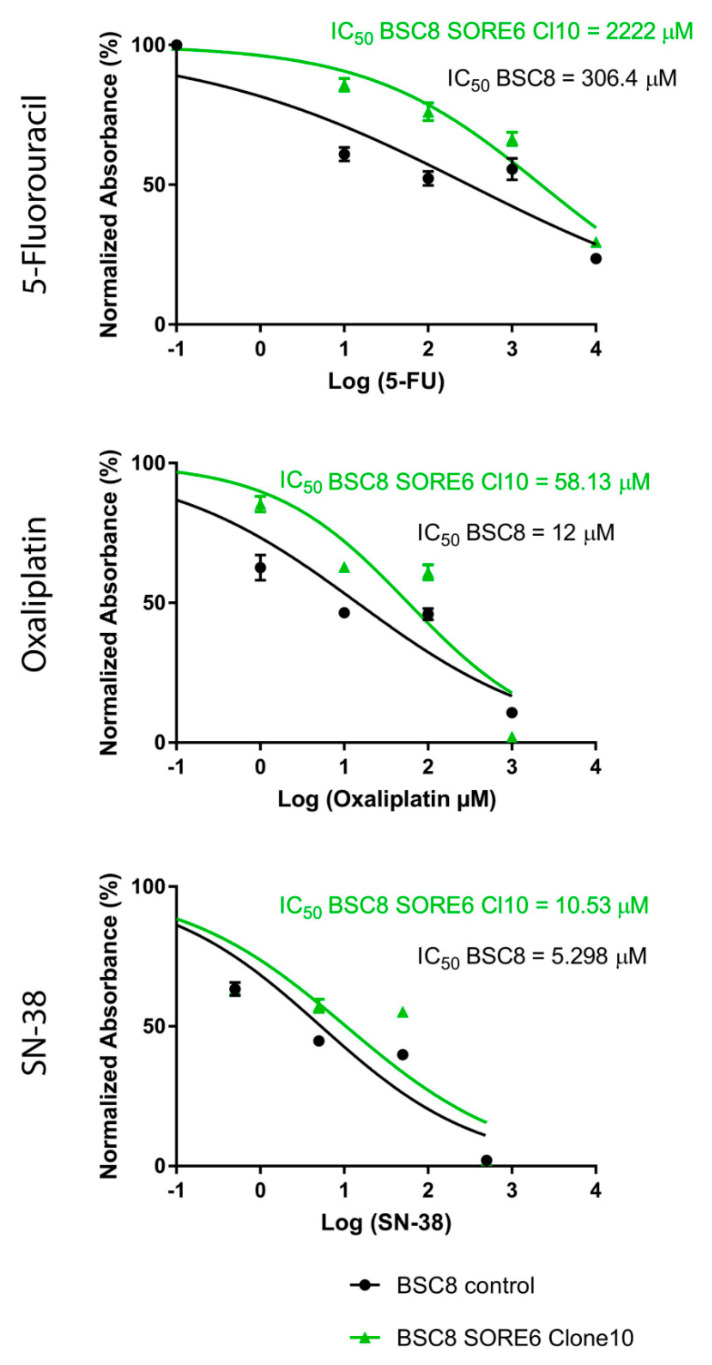
Inhibitory effect of 5-FU, SN-38, and oxaliplatin on cell growth. Cells were treated with increasing concentrations of 5-FU, SN-38, or oxaliplatin for 48 h, and cell viability was determined by the MTT assay. Each point represents the means ± SD. Experiments were performed in biological triplicate with similar results. The survival rate of the BSC8_SORE+/Clone 10 cells was significantly increased (by IC50) following treatment with 5-FU (seven times), SN-38 (five times) and oxaliplatin (6.8 times). Error bars = standard deviation.

We compared the functional properties of several cell clones obtained from a population of BSC8_SORE + cells. During the growth of the clones, we observed an increase in the degree of intraclonal heterogeneity of the cells, which could be judged by the level of GFP fluorescence (Figure 1D). In order to select the most malignant clone, we determined the degree of cell chemosensitivity to the effect of various concentrations of the most active cytostatics widely used in clinical practice (5-FU, oxaliplatin, and SN-38). The results for one of the clones compared to the original line are shown in the Figure 2. BSC8_SORE+/Clone 10 showed a seven-fold increase of the resistance to 5-fluorouracil (5-FU) treatment, five-fold to SN-38, and six-fold to oxaliplatin (Figure 2). The doubling time of the BSC8 line cells is 17 h, whereas in enriched stem cells (Clone 10) it is 23.3 h (1.3 times higher). Given that the chemoresistance is considered as one of the major properties of CSCs, further work done to characterize this clone.

### 2.3. Clone Formation under Normoxia and Hypoxia and Cell Migratory Activity Analysis

Next, we analyzed the expression of stem cell markers in the obtained cell line with the endogenic OCT4 and SOX2 expression. The levels of expression and the patterns of marker localization in the tumor cells of the original BSC8 cell line were similar to those of the BSC8_SORE+ line. There was no difference in the proportion of the cells expressing CD44, and its cytoplasmic expression as determined by staining was found in at least 40 percent of the cells. A significant increase in the proportion of LGR5 positive cells was noted in the BSC8_SORE+ cells. Weak cytoplasmic staining was found, when the antibodies to the OCT4 key embryonic stem cell transcription factor were used (Supplementary Figure S2).

We analyzed the functional characteristics of the isolated cell clones, such as clonogenic and migratory activity, as well as the mobility and chemoresistance. This analysis revealed that the cells do not differ in the level of clonogenic activity in a semi-liquid agar. Cells formed the same number of colonies, which also were identical in shape and size. The role of cultivation under hypoxic conditions was also analyzed. A larger number of cells expressing GFP on a section of colonosphere formed under hypoxic conditions can be noted. A looser structure of the colony in hypoxia was also noted (Supplementary Figure S3).

When growing BSC8_SORE+/Clone 10 cells on plastic, we observed an increase in the intraclonal cell heterogeneity. The levels of dsGFP expression visibly changed. During the flow cytometry analysis, we found the presence of 2 cell populations with 100× differences in the degree of fluorescence. The number of brightly fluorescent cells was twice as low as the number of cells with low level of GFP expression (Figure 1D).

**Figure 3:**
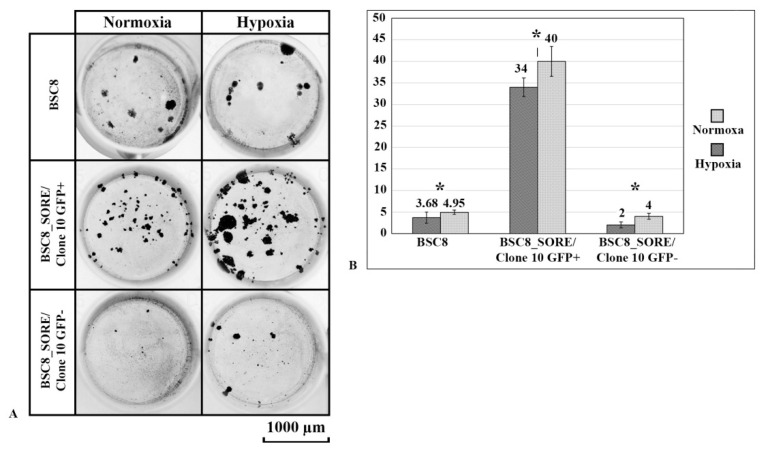
Clonogenic activity of the BSC8 tumor cells. (**A**). Formation of the cell colonies by the BSC8_SORE+/Clone 10/GFP+, BSC8_SORE+/Clone 10/GFP- cells and the original BSC8 cell line on plastic. An increase in the size of the colonies formed under the hypoxic conditions is observed. (**B**). Diagram of the number of colonies formed by the GFP+ and GFP- cells on plastic. A ten-fold increase in the clonogenic activity of the GFP+ cells is observed. All results are representative of three independent experiments. * *p* ≤ 0.05. Bars = standard deviation.

To find out how much the strengthening of GFP expression can reflect the “degree of stemness” of the cells selected, we isolated GFP+ cells by cell sorting. After plating the cells at the clonal density and culturing for two weeks, it was found that the BSC8_SORE+/Clone 10/GFP + cells exhibited increased clonogenic activity when cultured on plastic (Figure 3A). A ten-fold increase in the clonogenic activity of GFP+ cells obtained from BSC8_SORE+/Clone 10 cells was observed compared to the control samples (Figure 3B). We did not find differences in the clonogenic activity of “non-green” cells (GFP-) obtained from the BSC8_SORE+/Clone 10. In this case, the clonogenic activity was the same as in the cells of the original cell line (BSC8).

When cells were cultured under hypoxic conditions, there was a slight increase in the number of colonies, both in the original cells and in the SORE+ “stem-like” cells. We should also emphasize the increase in the size of the growing colonies, where the hypoxic conditions resulted in larger colonies (Supplementary Figure S3).

**Figure 4:**
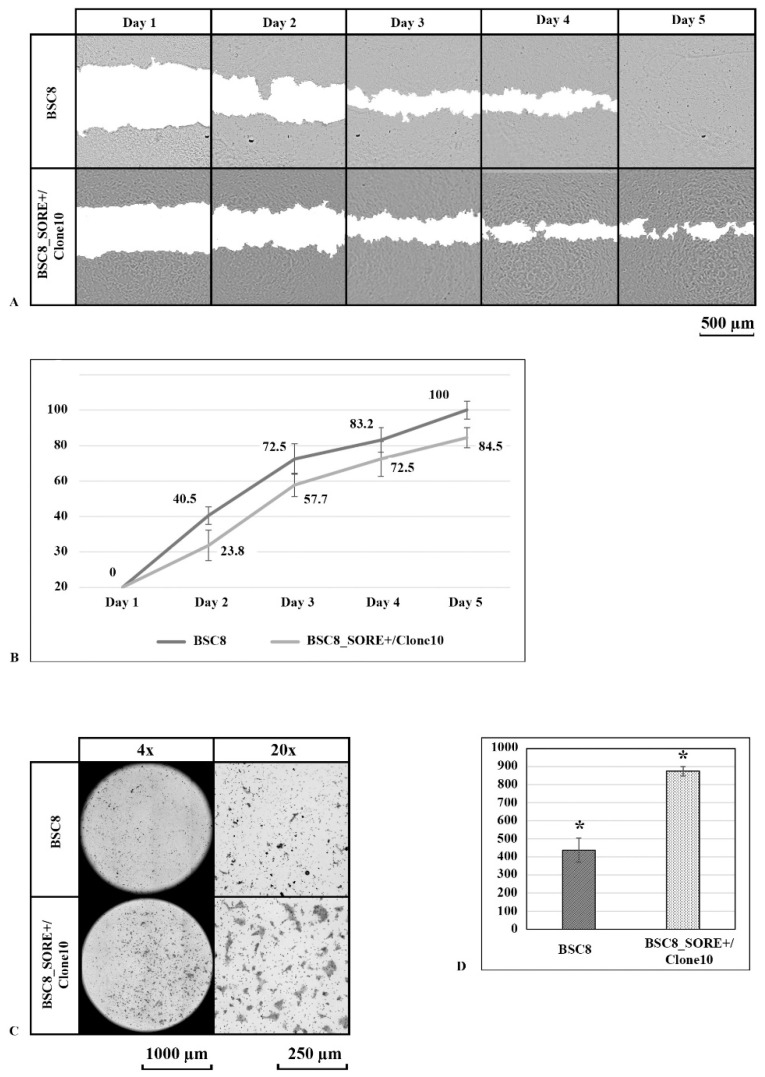
Comparison of the migratory activity of the “stem-like” BSC8_SORE+ cells and original BSC8 cells. (**A**). The growth of the SORE+ and original BSC8 cells during the “wound repair” test (original magnification 4×). (**B**). The intensity of wound healing by the SORE+ and original BSC8 cells. Complete closure (overgrowing) of the cell-free surface occurred on average after five days. A slight decrease in the migratory activity of the BSC8_SORE+ cells is observed. (**C**). Representative images from a Boyden chamber motility assay (original magnification 20×). (**D**). Quantitative analysis reflected the accelerated migration of the BSC8_SORE+ cells comparing with original BSC8 cells (437 ± 66.5 and 874 ± 25.5 clones per well, respectively; * *p* < 0.001). The results are averages with standard errors (*n* = 3 for each group).

Next, we analyzed the cell migratory activity by measuring the “wound repair” rate and by monitoring migration through the membrane of the Boyden chamber towards chemoattractant (represented by the medium with FBS). It was found that in comparison with and the original BSC8 line, the BSC8_SORE+ cell line was characterized by the somewhat reduced level of mobility—it took these cells more than five days for complete wound closure (Figure 4A,B). When analyzing migration activity through the pore towards the nutrient, the BSC8_SORE+ cells showed more than two times higher migration rate than the cell mobility of the original cell line. The large size of the formed colonies indirectly indicates a high clonogenic activity of the cells migrating through the membrane (Figure 4C,D).

### 2.4. Tumorogenic Activity of 5-FU Resistant Cells Compared to the Original Cells in the Xenograft Assay

**Figure 5:**
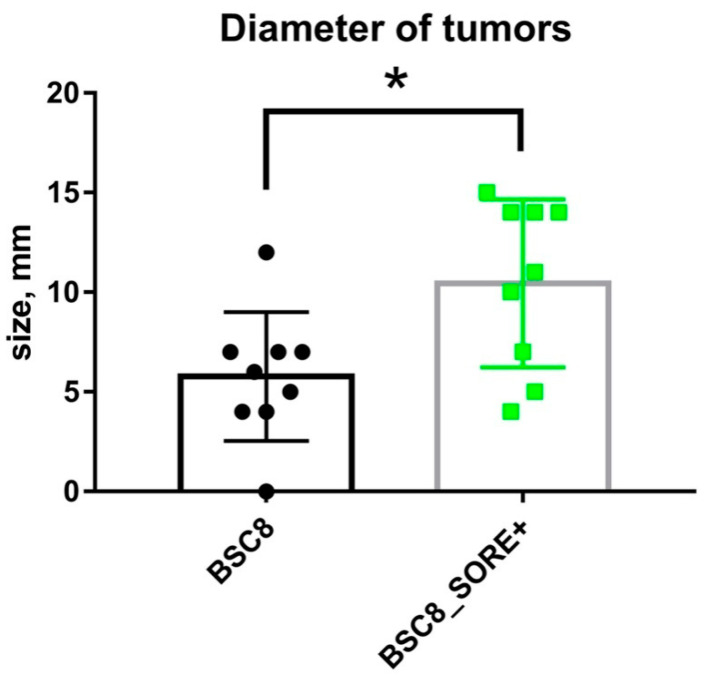
Comparison of the tumorogenic activity of the “stem-like” BSC8_SORE+ cells and original BSC8 cells, the sizes of tumors/measured in mice 40 days after the injection of BSC8_SORE+ cells and BSC8 are shown. Evaluation of the significance of the differences using the Student’s *t*-test showed that the sizes of tumors generated by BSC8 and BSC8_SORE+ cells significantly different on day 40, *p* < 0.01. * *p* < 0.05 for the difference.

CSCs are characterized primarily by their ability to regenerate new tumors in vivo. And although we observed properties similar to those of cancer stem cells, including increased cell chemoresistance, only an analysis of cell ability to form a tumor when injected into nude mice compared to the cells of the original cells could allow us to count them as CSCs-like cells. The results of the immunohistochemical staining of the original tumor and formed after BSC8 cells injection under the skin of an immunodeficient mouse withantibody CK-20; beta-catenin; CDX2; ALDH (data not shown) were similar, the intestinal origin of the tumor regeneraated in the mouse is not in doubt (Figure 5, Supplementary Figure S4).

In our xenograft model experiments, the cells were injected into the immune-deficient nude mice subcutaneously in the thigh area, a single-cell suspension of 500,000 cells in 100 µL of Hanks’ solution. In the experiment, 10 nude mice were taken (one died), the size of the tumors measured on day 40 after the injection of BSC8_SORE+ cells (left thigh) and BSC8 (right thigh) is shown in the Figure 5 and Supplementary Figure S4. Due to the limited number of Nude mice injections were given under both legs. Thus, we compared the ability of injected cells to regenerate new tumor and the sizes of them. Tumorogenic activity was evaluated by measuring the tumor diameters at the time of 20, 30 and 40 days from the day of injection. Thus, it is shown that the enrichment of the BSC8population with the help of a reporter containing a promoter of the nanogene carrying a binding site for the Oct4/Sox2 complex, contributes to an increase in their tumorogenicity. We believe that our results show the activity of expression of POU-domain transcription factors in the stem-like component of the tumor population and characterize this family of proteins as a marker of cancer stem cells and a potential target for genetic cancer therapy. The characteristics of the BSC8_SORE+ cells we present give us reason to believe that they are similar to cancer stem cells, and further in the text we allow ourselves to call them a subpopulation of CSC-like cells.

### 2.5. The Transcriptomic Landscape of the SORE+ Cells

**Figure 6:**
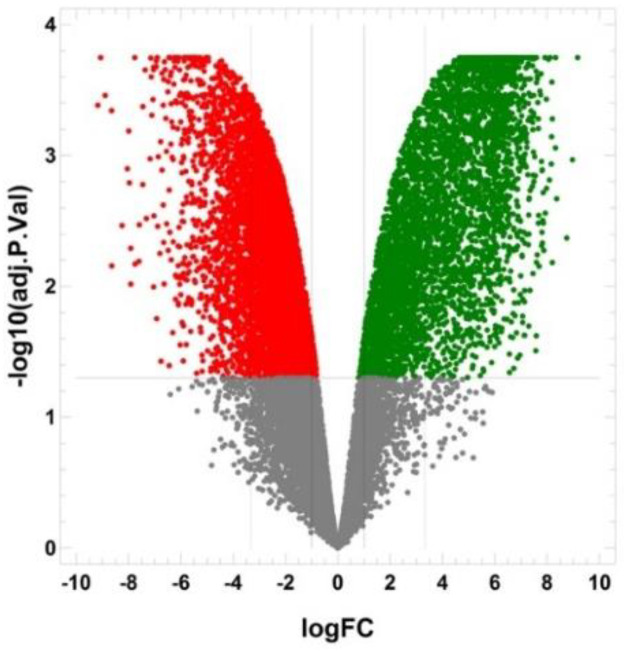
Volcano plot for differential gene expression. BSC8_SORE+ vs BSC8 cells as control. X axis –Log2 fold change for BSC8_SORE+ vs BSC8 cells. Y axis –log10 of adjusted *p*-value for fold change. Green dots are significantly up-regulated genes, red dots are significantly down-regulated genes.

BSC8_SORE+ cancer stem cell-like cell line demonstrated differential expression of 12831 genes compared to BSC8 control line. Of these genes, 5973 were up-regulated and 6451 were down-regulated (Figure 6)

**Figure 7:**
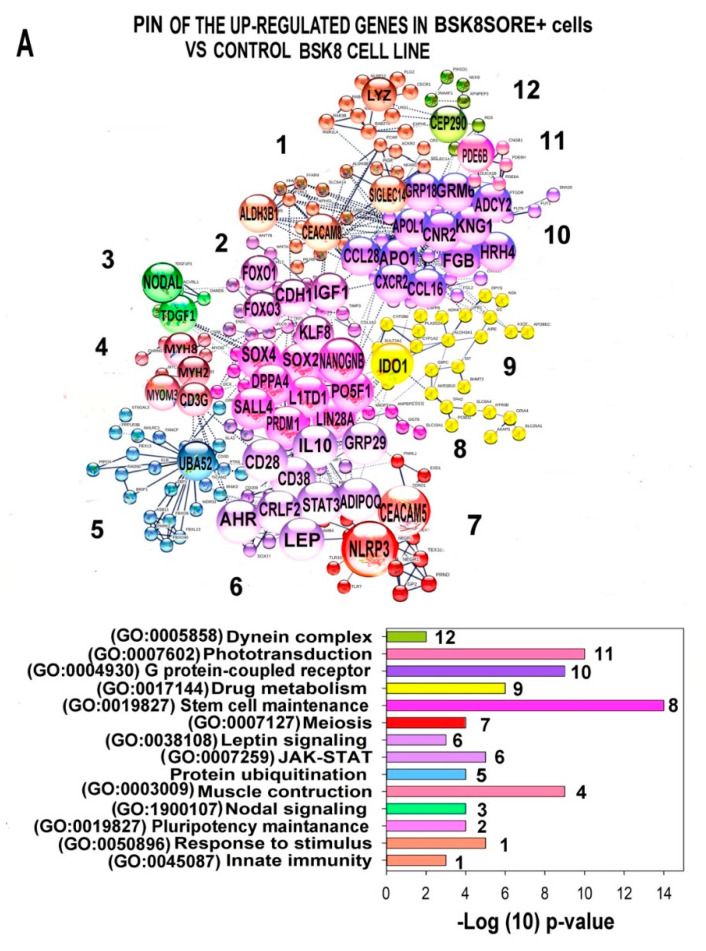

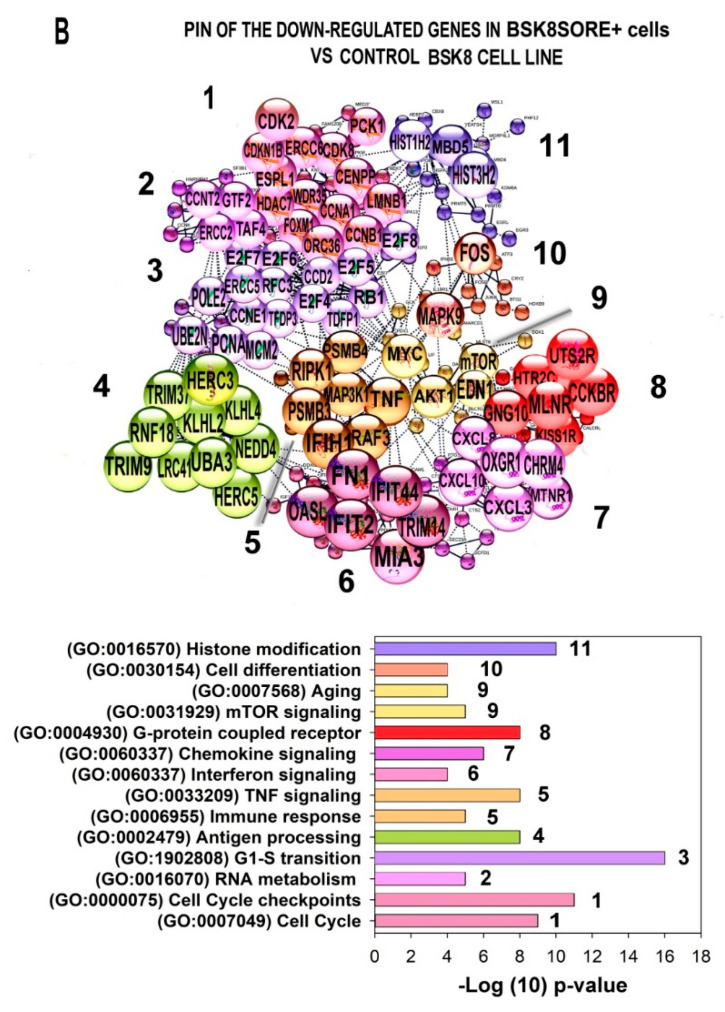
The most connected components of protein interaction networks of the up- (**A**) and down- (**B**) regulated genes in the stem cell-enriched subpopulation of the CSC-like BSC8_SORE+ cell line vs. the original population of the BSC8 cell line. The network was constructed using the STRING server with the highest interaction confidence. Clusterization was provided by k-means clustering. Hub proteins, that is, the ones having more than five connections, are shown as large buttons; node proteins are shown as plain small buttons. Fold expression difference is not less than 10-fold. GO biological processes that enrich for clusters from the up- and down-regulated networks with the highest significance are indicated in bar charts under the networks. Cluster numbers coincide with the numbers of bars on the charts.

To identify the most prominent functional effects, we first constructed the protein-protein interaction network (PIN) for proteins encoded by the most up- or down-regulated genes with the expression difference of more than 10-fold and with the best interaction confidence (>0.9). The network was constructed using the String server (https://string-db.org/, accessed on 12 August 2020). Next, we extracted the whole connected component from the induced and inhibited networks and performed k-means clustering using the same server. Figure 7 and Supplementary Figure S5 show versions with short and extended legends of the induced and the inhibited PINs. It is clearly seen that the up-regulated and down-regulated networks contain the large tightly linked clusters involved simultaneously in several functions that confirm a modular pattern of transcriptome changes. The pattern of gene function distribution among various clusters indicated that in accordance with the experimental data, BSC8_SORE+ demonstrated clear manifestations of drug resistance, pluripotency, and decreased proliferation rate. Below, we provide a detailed description of the main clusters from the up- and down-regulated PINs. The regulators containing more than five bonds, we designated as hub proteins.

### 2.6. Characterization of the PINs and Enrichment of Gene Modules for Genes Differently Expressed in BSC8_SORE+ vs. BSC8

The PIN for the up-regulated genes is shown at Figure 7A and Supplementary Figure S5A. Gene expression fold difference for the up-regulated genes from this PIN is listed in Supplementary Table S1. The PIN for the induced genes contained 12 clusters. The largest of these clusters were related to the regulation of pluripotency and pathways maintaining pluripotency. The main hubs of pluripotency clusters were the regulators of stem cell differentiation and maintenance, including NANOGNB (PPIDR_PONDR-FIT_ = 62.8%), POU5F1 (39.4%), LIN28A (46.4%), SALL4 (43.6%), SOX4 (82.3%), SOX2 (95.3%), LIN28A (47.4%), FOXO3 (81.9%), KLF8 (60.7%), and IGF1 (47.7%). Additionally, manifestations of stemness were seen from the induction of Nodal signaling responsible for regulation of the morphogenesis, migration, and epithelial to mesenchymal transition and from the gametogenesis cluster. The induced network also contained several clusters that do not participate in the pluripotency regulation directly but can promote stem cell survival. These clusters were implicated in innate immunity [38], ubiquitin protein degradation [39], and phototransduction signaling that mediate neuronal morphogenesis [40]. The PIN for the down-regulated genes contained 12 clusters. The induced PIN also revealed the features of drug resistance. It can be seen from the cluster of drug metabolism through cytochrome p450 pathway (hubbed by IDO1), leptin signaling, and Toll-like receptors. The induction of these clusters both confirmed our experimental results indicating a higher drug resistance (specifically, for 5-fluorouracil), and also showed which particular pathways confer this property [41,42].

The PIN for the down-regulated genes is shown in Figure 7B and Supplementary Figure S5B. The expression fold difference for the down-regulated genes from this PIN is shown in Supplementary Table S2. The PIN for the down-regulated genes contained 11 clusters and also showed manifestations of stemness. For example, the clusters of aging (the main hubs are AKT (PPIDR_PONDR-FIT_ = 18.3%), mTOR (23.6%), and EDN1 (44.3%) and differentiation (the main hub is MAPK9 (PPIDR_PONDR-FI T_ = 18.4%) point to dedifferentiation (Figure 7B, Supplementary Figure S5B). The clusters of cell cycle regulation evidence of slow cell cycling. This feature is a well-known manifestation of stemness [43] Many hubs of these clusters were classical cell cycle regulators of E2F, cycline (CCN), and CDK, RB, and PCNA families. Another important cell cycle-related cluster was implicated in chromatin modification via histones (cluster 1, the main hubs were HIST1H2 (PPIDR_PONDR-FIT_ = 78.4%) and HIST3H2 (PPIDR_PONDR-FIT_ = 44.4%). The clusters of immune response and inflammation via interferon signaling, chemokines and TNF (PPIDR_PONDR-FIT_ = 12.4%) in the PIN of down-regulated genes suggest a decreased activity of pro-inflammatory signaling. Since pro-inflammatory signaling stimulates stem cell differentiation [44], the decrease of the activity of this pathway also points to the cell dedifferentiation.

Therefore, the functional picture provided by PIN clusters for the inhibited and induced genes indicated that compared to the BSC8 control cells, BSC8_SORE+ cells demonstrate manifestations of the pluripotency and drug resistance. The unexpected result was the induction of genes participating in the leptin signaling and phototransduction. BSC8_SORE+ cells also showed the coordinated down-regulation of signaling involved in the cell cycle regulation, and phase transition, inflammation, aging, and differentiation, which complements the data obtained with the induced network.

### 2.7. Gene Module Analysis

**Figure 8:**
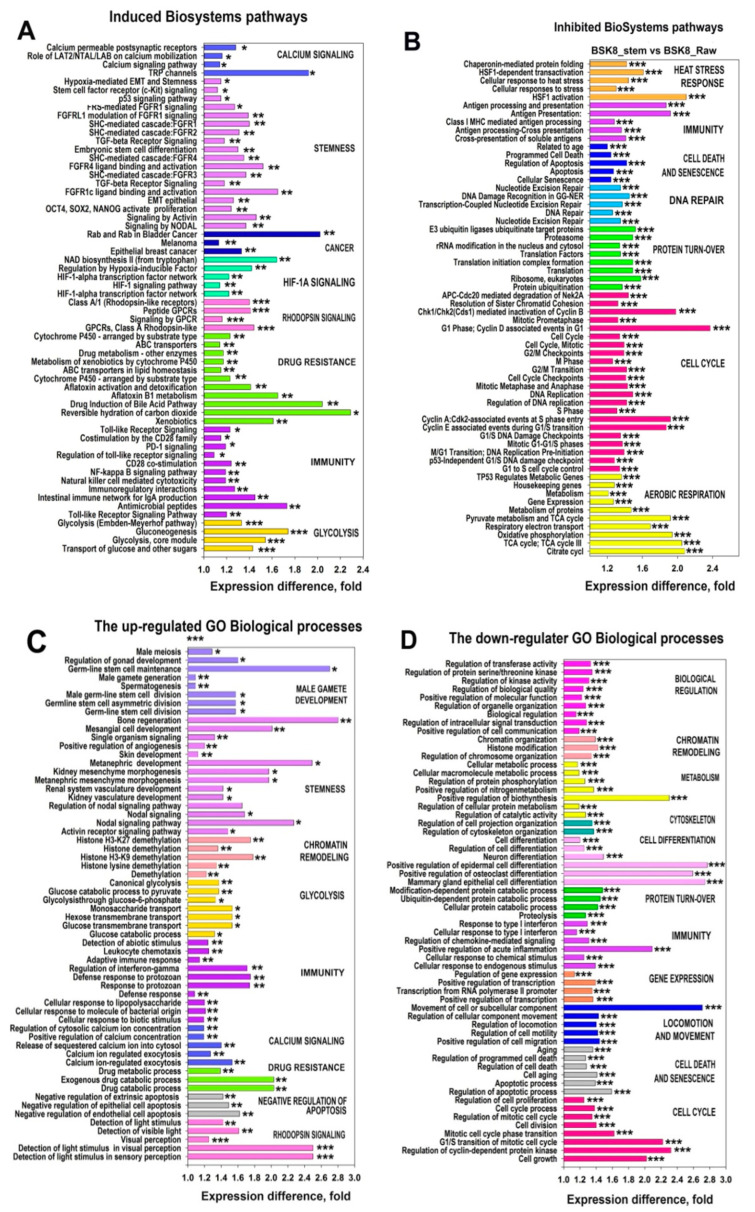
60 most significantly up-regulated (**A**,**C**) and down-regulated (**B**,**D**) GO Biological processes and NCBI BioSystems pathways in the CSC-like cell line vs the original population of the BSC8 cell line. Gene modules with similar functions are marked with the same color. * *q* < 0.05; ** *q* < 0.01; *** *q* < 0.001.

To obtain a transcriptome-wide picture and to find out whether the discovered effects were manifested globally, we performed Gene Ontology (GO) and Cancer BioSystems analysis of functional gene modules (by averaging gene expression). Sixty most significantly up- and down-regulated modules are shown in Figure 8A–D and Supplementary Tables S3–S6. The results of this analysis provided important support to the PIN analysis data. For example, similar to the trends found by the analysis of PIN clusters, the substantial number of the induced modules from both databases were related to the pluripotency, including signaling via NODAL (PPIDR_PONDR-FIT_ = 13.3%) and activin (branches of the TGF-β pathway), and signaling via Yamanaka factors (POU5F1 (PPIDR_PONDR-FIT_= 39.4%), NANOG (62.8%), and SOX2 (95.3%). Additionally, several modules were related to TGF-β, c-Kit, and FGF signaling and to EMT and stemness, as well as to male gametogenesis and development (Figure 8A,C, Supplementary Tables S3 and S5). A large number of the up-regulated modules were also involved in the response to various stressors and stimuli, such as drug resistance and antimicrobial immunity. Importantly, gene module analysis confirmed the activation of phototransduction and leptin signaling pathways. The new features revealed by the gene module analysis were the up-regulation of cancer-related modules including calcium and HIF1α signaling, pathways of glycolysis, cancer, and negative regulation of apoptosis (Figure 8B,C).

In accordance with the PIN analysis results, the down-regulated gene modules were related to the cell growth, proliferation, cell cycle regulation and phase transition, aging, differentiation, chromosome organization, chromatin remodeling, immune response (including interferon signaling, inflammation, antigen processing, and cytokine signaling), and RNA metabolism (Figure 8B,D, Supplementary Tables S4 and S6). The new down-regulated modules were represented by several pathways related to apoptosis and numerous pathways associated with the protein metabolism. Therefore, the gene module analysis results confirmed that the effects identified for the most deregulated genes by PIN analysis apply to the entire transcriptome.

To verify the resistance of the BSC8_SORE+ cells to 5-fluorouracil via the in silico analysis, we used CDRgator (Cancer Drug Resistance navigator) [45]. CDRgator (http://cdrgator.ewha.ac.kr, accessed on 15 September 2020) is a database that unifies data on the drug resistance gene signatures for about 1000 cancer cell lines and cancer cells of resistant patients (for about 30 drugs). Of all gene signatures, we selected ones involved in the regulation and responsible for the 5-fluorouracil resistance of colorectal carcinoma. As a result, we obtained three gene signatures (30, 35, and 600 genes) and compared them with our gene expression data using binomial test for the statistical significance evaluation (Supplementary Table S7).

### 2.8. Intrinsic Disorder Predisposition of Proteins Encoded by Genes Differently Expressed in the BSC8_SORE+ Cells vs. BSC8 Control Cells

High binding promiscuity and capability to interact with various often unrelated partners serve as characteristic features of intrinsically disordered proteins (IDPs) or hybrid proteins containing ordered domains and intrinsically disordered protein regions (IDPRs) [46,47,48,49,50,51,52]. IDPs/IDPRs are capable of forming static, semi-static, dynamic, or fuzzy complexes [46,47,48,50,51,52], be engaged in semi-static and dynamic polyvalent interactions [53,54], bind partners with high specificity and low affinity [55], and fold (at least partially and often differently) at interaction with different specific partners [56,57,58,59,60,61,62]. High structural and functional heterogeneity of IDPs/IDPRs and their complexes [50,52] defines protein structure-function continuum, where a dynamic conformational ensemble of a given protein includes multiple conformational, inducible, and functioning proteoforms (i.e., different molecular forms in which the protein product of a single gene can be found) characterized by a broad spectrum of structural features and possessing various functions [63].

Since IDPs/IDPRs are known to play crucial roles in PIN regulation, and since many IDPs/IDPRs serve as hub proteins [61,64,65,66,67,68], we also evaluated intrinsic disorder predispositions of the proteins encoded by the up- and down-regulated genes with very high significance in the CSC-like BSC8_SORE+ cell line (Supplementary Tables S8 and S9). The genes encoding these proteins are also listed in Supplementary Tables S1 and S2.

**Figure 9:**
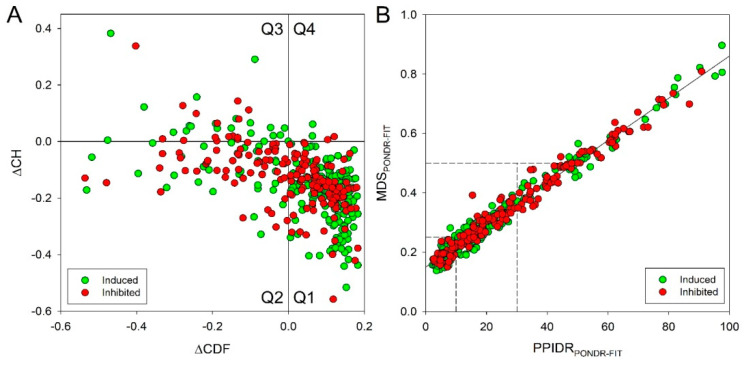
Global intrinsic disorder predisposition of 219 induced (green circles) and 175 inhibited proteins (red circles) in the CSC-like BSC8_SORE+ cell line vs. the original population of the BSC8 cell line. (**A**) CH-CDF plot analysis. Quadrants are numbered in a clockwise direction starting with Q1 in the lower right and ending with Q4 in the upper right. The following characterize proteins within each quadrant: Q1 (lower-right quadrant)—proteins predicted to be ordered by both predictors, Q2 (lower-left quadrant)—proteins predicted to be ordered by CH but disordered by CDF, Q3 (upper-left quadrant)—proteins predicted to be disordered by both predictors, and Q4 (upper-right quadrant)—proteins predicted to be disordered by CH and ordered by CDF. (**B**) Correlation of the mean disorder scores (MDS) and PPDRs based on the results of the PONDR^®^ FIT analysis. Dashed lines represent boundaries separating ordered (MDS < 0.25, PPDR < 10%), moderately disordered (0.25 ≤ MDS < 0.5, 10% ≤ PPDR < 30%), and highly disordered proteins (MDS ≥ 0.5, PPDR ≥ 30%).

Figure 9A represents results of the CH-CDF analysis of these two protein sets. This approach allows predictive classification of proteins based on their position within the CH-CDF phase space into structurally different classes, such as ordered proteins, proteins with compact disorder/hybrid proteins and proteins with extended disorder [69,70,71,72]. This analysis revealed that among the 219 induced proteins, 21 proteins is expected to be highly disordered, 32 proteins might have a molten globular or hybrid structure, and 164 proteins are mostly ordered. Curiously, 175 inhibited proteins showed a bit different disorder status, with 15, 51, and 107 being expected to behave as native coils/native pre-molten globules, native molten globules/hybrid proteins, and ordered proteins, respectively. In other words, 24.2% of the induced proteins and 37.8% of the inhibited proteins are expected to have high levels of intrinsic disorder.

Based on the peculiarities of the results generated by the per-residue disorder predictors, such as content of disordered residues (or percent of predicted intrinsically disordered residues, PPIDR) and mean disorder score (MDS), proteins are typically grouped into three categories: highly ordered (PPIDR = 0–10% or MDS = 0.00–0.25), moderately disordered (PPIDR = 10–30% or MDS = 0.25–0.50), and highly disordered (PPIDR greater than 30% or MDS greater than 0.50). Obviously, the MDS value calculated for a given protein is not directly related to its PPDR value (e.g., theoretically, a protein with the PPDR of 100% might have the MDS ranging from 0.5 to 1.0; whereas a protein with the PPDR of 0% might have any MDS < 0.5). Figure 9B shows the MDS vs. PPDR plot generated for the induced and inhibited proteins in the CSC-like BSC8_SORE+ cells based on the results of their analysis by the meta-predictor of intrinsic disorder, PONDR^®^ FIT, which is slightly more accurate than any of its component predictors. Note, that the results of the analogous analysis using some of the components of the PONDR^®^ FIT (PONDR^®^ VLXT, PONDR^®^ VL3, PONDR^®^ VSL2, IUPred_L and IUPred_S) are provided in Supplementary Materials (Supplementary Tables S8 and S9).

Overall, this PONDR^®^ FIT-based analysis indicated that the majority of the induced and inhibited proteins are either moderately or highly disordered. In fact, in the induced set, 68 (31.1%), 96 (43.8%), and 55 proteins (25.1%) have PPIDR scores of PPIDR < 10%, 10% ≤ PPIDR < 30%, and PPIDR ≥ 30%, respectively, indicating that 68.9% of these proteins are either highly or moderately disordered. Similar picture is observed, when these proteins are grouped based on their MDS values: 95 (43.4%), 94 (42.9%), and 30 (13.7%) have MDS < 0.25, 0.25 ≤ MDS < 0.5, and MDS ≥ 0.5, respectively. Therefore, based on their MDS values, high or moderate levels of disorder is found in 124 (56.6%) induced proteins. Application of the analogous classification to inhibited proteins indicated that 39 (22.3%), 71, (40.6%), and 65 (37.1%) of them have PPIDR < 10%, 10% ≤ PPIDR < 30%, and PPIDR ≥ 30%; and 52 (29.7%), 92 (52.6%), and 31 (17.7%) of these proteins have MDS < 0.25, 0.25 ≤ MDS < 0.5, and MDS ≥ 0.5. In other words, based on their PPIDR/MDS values, 77.7%/70.3% of inhibited proteins belong to moderately or highly disordered category. Figure 9B also gives us a possibility to select the most disordered proteins in the sets, as proteins possessing MDS ≥ 0.5 and/or PPDR ≥ 30%. This analysis indicated that 55 (25.1%) and 65 (37.1%) of the induced and inhibited proteins satisfy these criteria. Even using PPIDR ≥ 50% as the most stringent criteria for the protein to be classified as highly disordered, we found that 26 (11.9%) and 29 (16.6%) of the induced and inhibited proteins belong to this category.

Curiously, although it seems that the inhibited proteins are a bit more disordered than the induced, ones, the Top-10 induced proteins showed higher levels of disorder and the Top-10 inhibited proteins. In fact, PPIDR scores of 10 most disordered proteins in the induced set ranged from 97.6% to 76%, whereas in the inhibited set, 10 most disordered proteins were characterized by the PPIDR scores were somewhat lower ranging from 90.8% to 67.1%. Finally, the potential relation of intrinsic disorder to the functionality of induced and inhibited proteins was stressed out by adding the corresponding PPIDR values to the proteins discussed in the previous sections and in Discussion section below. Importantly, most of these proteins are either moderately or highly disordered, indicating that the intrinsic disorder is needed for the function of proteins associated with stemness of CSCs.

## 3. Discussion

In this study, we obtained a line of the colon adenocarcinoma stem-like cells. The selection was based on the activation of reporter genes driven by the binding of the SOX2 and OCT4 endogenous stem cell factors. Selected cells were characterized by increased clonogenic and migration activity, and also showed increased resistance to cytostatic drugs. Using bioinformatics methods, we analyzed the levels of gene expression, combining them into functional groups, followed by the analysis of their roles in the maintenance and functioning of tumor stem cells. We also checked the intrinsic disorder status of the corresponding proteins.

Stem cell renewal can be achieved by symmetric division, as well as asymmetric division [73,74]. Symmetric division of stem cells is a highly conservative and precisely regulated process of division resulting in formation of two cells, which differ in potentials, morphology, and functions [75]. During long-term cultivation, the population of GFP+ cells acquire heterogeneity, which we explain by the differentiation of the part of the cells. FACS sorting of this heterogeneous population allowed us to obtain cells with low and high clonogenic activity. The clonogenic activity of the BSC8_SORE/Clone 10/GFP+ cells were 10 times higher than the activity of the cells from the original BSC8 line. This confirmed that the GFP+ cells had stem properties.

It is assumed that only cells with unlimited proliferation ability are capable of forming clones both in vitro and in vivo in immunodeficient mice [76,77]. An increase in the clonogenicity of tumor cells under the influence of various factors (for example, activation of the expression of embryonic transcription factors) is interpreted as an increase in the “stemness” and, hence, malignancy potential [76]. The transcriptome analysis revealed high levels of expression of a number of stem factors involved in the ensuring both normal and tumor stemness. Next, we described some of these factors in more detail.

Due to the growing interest in tumor stemness, a lot of results have been accumulated concerning the expression of OCT4 variants in tumors of different localization, and although OCT4A expression is limited to embryonic cells and embryonic carcinoma cells, it has been noted that OCT4B is expressed in human somatic stem cells, tumor cells, adult tissues, and pluripotent cells [78,79,80,81,82]. The pluripotency is an important feature of the CSCs that promotes the self-renewal and chemoresistance. Preservation of embryonic stem cell (ESC) pluripotency under the different pathophysiological conditions requires a complex interaction between different cellular pathways, including those involved in the homeostasis and energy metabolism. However, the exact mechanisms that support CSC pluripotency remain unclear. It seems that the molecular pathway of the self-renewal in normal stem cells is the same as that of CSCs in tumor [83]. Many self-renewal regulatory factors, such as OCT4, SOX2, BMI, and NANOG, are expressed in human malignant tumors, and they play an important role in carcinogenesis.

The ability of CSCs to self-maintain, being a common feature with ESCs, provides CSCs with the ability to form metastatic tumors and also contributes to the activation of some embryonic mechanisms to protect cells from the effects of chemotherapy and autoimmune aggression. The expression of many oncofetal and testicular antigens generally correlates with a negative clinical prognosis of cancer. We will discuss only some examples of proteins characteristic for CSCs, which were identified during comparative analysis of BSC8_SORE+ and the original BSC8 cell line transcriptomes.

Among the genes with an increased level of expression in the BSC8_SORE+ cells, we should note a few key factors of ESC maintenance and some factors involved in the chromatin remodeling, such as: SOX2 (PPIDR_PONDR-FIT_=95.3%), SOX11 (77.6%), SALL4 (43.6%), PRDM14 (23.8%), L1TD1 (55.4%), LIN28B (47.4%), POU5F1 (39.4%), DPPA2 (36.9%), and NANOG (62.8%). The high level of expression of these factors in tumors always indicates a high aggressiveness of the process and serves as negative prognostic factors. Increased activity of core embryonic transcription factors—OCT4/POU5F1 (PPIDR_PONDR-FIT_ = 39.4%), SOX2 (95.3%), and NANOG (62.8%)—in the BSC8_SORE+ cells is probably involved in implementing the CSC self-maintenance and pluripotency properties in the same way as described for embryonic stem cells.

As an example of a protein with an increased level of expression, we should mention another important transcription factor encoded by the oncofetal gene, SALL4 (PPIDR_PONDR-FIT_ = 43.6%) [84,85]. Along with other solid tumors, its increased expression is described in colon cancer cells [83]. An increased expression of co-receptor for nodal signaling pathways TDGF-1/CRIPTO-1 (PPIDR_PONDR-FIT_ = 10.6%) is observed in the BSC8_SORE+ cells, also previously described as a factor of unfavorable prognosis. This factor plays an important role in the maintenance of stem cells and in the activation of cell metastatic abilities by increasing mobility, increased MMP2 (PPIDR_PONDR-FIT_ = 2.42%) expression, induction of EMT transition and chemokine receptor CXCR4 [86,87,88,89,90].

The results of numerous studies confirm the role of a complex of pathways involved in the cell chemoresistance in the biology of CSCs and the realization of their stem potential. According to the transcriptome analysis, the BSC8_SORE+ cells express the elevated levels of ABCB5 (PPIDR_PONDR-FIT_ = 8.04%). Its expression allows tumor cells to form a multidrug resistance (MDR) phenotype enabling the resistance to 5-FU chemotherapy [91,92]. A number of studies have shown the role of ABCB5 in the implementation of resistance and functioning of cancer stem cells of such types of such solid tumors as that of the oral cavity and colon cancer [93,94]. The CYP1A2 (PPIDR_PONDR-FIT_ = 5.43%) involved in the phase 1 excretion of xenobiotics, is expressed at elevated level in the BSC8_SORE+ cells. IDO1 (Indoleamine 2,3-dioxygenase) is a key regulator of the activity of these enzymes. IDO1 metabolites (in particular tryptophan metabolites) are capable of inhibiting the functions of cytotoxic T lymphocytes, leading to local immunosuppression [95,96,97,98].

The activity of co-transporters and enzymes can significantly reduce the intracellular concentration of drugs. The combination of the activity of DNA repair systems together with the violation of the restriction points of the cell cycle leads to the CSC survival accompanied by a gradual accumulation of new mutations. As for DNA repair, it should be noted that the increased activity of a number of auxiliary proteins, such as NEK8 (PPIDR_PONDR-FIT_ = 12.4%), taking part in the activity of RAD51-dependent DNA repair (NEK8 regulations of DNA damage-induced RAD51 foci formation and replication for protection) were also shown in the BSC_SORE+ cells.

An important role in ensuring cytostatic resistance is the retention of cells in a state of mitotic rest (G0 phase). “Dormant” tumor cells are not sensitive to most cytostatic agents, as well as are more resistant to adverse microenvironment conditions of distant metastasis formation [99]. One of the distinctive features of the BSC8_SORE+ cells is the cell cycle slowing down and the cell metabolism inhibition. According to experimental data, the BSC8_SORE+ cell cycle duration is 23.3 h, which is 1.3 times longer than original BSC8 cells have. Two gene clusters are implicated in the cell cycle regulation. Hubs (E2F4-8 (PPIDR_PONDR-FIT_ = 50.6%, 39.9%, 24.9%, 45.2%, and 34.5%, respectively), CCNE (26.6%), POLE (7.02%), PCNA (15.7%), and RB1 (28.0%), and other hubs (CCNA (PPIDR_PONDR-FIT_ = 7.96%), CCNB1 (36.0%), CDKN1B (76.8%), CENPP (17.0%), CDK8 (28.7%), and FOXM1 (61.6%) are reflected in the lower level of mitotic activity. At the same time, we can observe the inhibition of protein metabolic activity. Signs of metabolic deprivation are also consistent with the cell cycle slowing down. It is known that the cell cycle slowing down is one of the key CSC properties and can lead to an increased resistance to cyclo-dependent cytostatics (5-FU and SN-38) [100]. The selection of a cell population with a low cycling rate is one of the methods of enrichment of highly malignant cells. The selected cells correspond to the CSC criteria and exhibit the increased chemoresistance, clonogenic activity, migratory activity, and also express various extracellular matrix-degrading enzymes [75,101,102].

One of the most important aspects of the tumor cell functioning is the mechanisms of local immunosuppression. The inhibition of the cluster of proteins involved in interferon signaling (such as IFIT2 (PPIDR_PONDR-FIT_ = 22.7%), IFIT44 (3.60%), TRIM14 (18.8%), etc.), the TNF-signaling (such as TNF (PPIDR_PONDR-FIT_ = 12.4%), RIPK1 (27.6%), MAP3K1 (47.9%), IFIH1 (22.1%), TRAF3 (18.7%), etc.) were observed in the BSC8_SORE+ cells. There is an increased IL-10 (PPIDR_PONDR-FIT_ = 18.5%) expression level that can lead to a variety of immunological reactions including suppression of cytokine synthesis, antigen presentation, and suppression of CD4+ T lymphocytes. STAT3 (PPIDR_PONDR-FIT_ = 15.3%) expression activation observed according to the transcriptome analysis (noted previously) can be caused by the IL-10 expression.

The expression levels of chemokine receptors and their ligands, such as CXCR2, CXCL14, CCL16, CXCL17, CCL28, and CCL22 (with the PPIDR_PONDR-FIT_ of 13.1%, 32.3%, 24.2%, 72.3%, 35.4%, and 18.3%, respectively) were also increased in BSC8_SORE+ cells. At the same time, there was the repression of CXCL1, CXCL3, CXCL8, CXCL10, and CXCL11 (PPIDR_PONDR-FIT_ of 24.5%, 25.2%, 16.2%, 24.5%, and 23.4%, respectively). In physiological conditions, chemokines participate in the directed movement of immune cells (in particular lymphocytes). Their role in the development of tumor cell metastasis to lymph nodes and distant organs is assumed to be guiding [103,104]. There are also papers showing chemokines participation in the stem cell functioning processes. Overexpression of CCL28 enhances cell proliferation, as well as their migration and clonogenic properties [105]. According to the authors, these manifestations occur due to the MAP kinase cascade activation.

The aforementioned CXCR2 overexpression is consistent with the literature data stating that the CXCR2 overexpression on the surface of tumor cells is a marker of poor prognosis for some types of cancer [106,107,108,109]. The use of CXCR1/2 oral inhibitors leads to the inhibition of metastasis to the liver of colon cancer due to the inhibition of neovascularization and increased apoptosis of tumor cells [110]. The importance of angiogenesis for tumor growth and spreading is undeniable. In the obtained stem cells, we observed the increased expression of angiopoietins ANGPT2 and ANGPT4 with the PPIDR_PONDR-FIT_ of 8.07% and 32.8%, respectively, which are the TIE2 receptor ligands on the surface of endothelial cells [111,112]. Their expression promotes VEGF-independent tumor neovascularization. According to the literature, they are negative prognostic markers [113,114,115,116,117].

Finally, our bioinformatics analysis indicated that a significant portion of up- and down-regulated proteins in the CSC-like BSC8_SORE+ cells are moderately or highly disordered. In fact, induced and inhibited proteins contain variable levels of intrinsic disorder, and their PPIDR_PONDR-FIT_ values ranges from 2.21% to 97.6% and from 2.65% to 90.8%, respectively. Consideration of the presence of intrinsic disorder in proteins associated with the stemness of CSCs provides an important angle for better understanding of their functionality. In fact, high disorder content in many stemness-associated proteins can be related to the presence multiple posttranslational modification (PTM) sites and numerous isoforms generated in these proteins by alternative splicing, as well as it can define their binding promiscuity and ability to undergo binding induced folding at interaction with specific partners. Such disorder-based structural and functional heterogeneity of human proteins associated with CSC stemness is in agreement with the well-established fact that IDPs or hybrid proteins containing ordered domains and IDPRs are typically involved in recognition, regulation, and cell signaling [58,61,118,119,120,121,122,123,124,125,126,127,128,129,130,131,132], and are commonly found among proteins related to the pathogenesis of various human diseases [123,133,134,135,136,137,138,139,140,141]. These findings are also in line with the previously reported roles of intrinsic disorder in reprogramming transcription factors (also known as Yamanaka factors, such as SOX2, OCT3/4 (POU5F1), KLF4, and c-MYC, and the Thomson factors, such as SOX2, OCT3, LIN28, and NANOG), overexpression of which leads to the transformation of terminally-differentiated somatic cells into the induced pluripotent stem (iPS) cells [142].

## 4. Materials and Methods

### 4.1. Obtaining the Primary Cell Line

The material for this study was obtained on the basis of the patients’ informed consent. The ongoing research received the ethical approval of the ethics committee of the St. Petersburg Clinical Research and Practical Center of Specialized Types of Medical Care (Oncologic). Tumor material was obtained during the planned surgical treatment of 12 patients. Tissue fragments were transported to the laboratory within 6–12 h after surgery in saline solution.

The cells were isolated from the primary tissue based on the recommendations of Yu et al. [143] and according to our previous study [144]. Each sample was cut into pieces of approximately one cubic millimeter in size and incubated in 10-fold (by volume) excess of trypsin (Gibco, Gaithersburg, MD, USA) overnight in a refrigerator at 4 °C, followed by an hour incubation at 37 °C. The action of trypsin was inhibited by RPMI medium with 10% FBS (fetal bovine serum). This suspension of disintegrated cells was centrifuged to collect the cells (150 g for 5 min). The cells were then resuspended in Roswell Park Memorial Institute (RPMI) 1640 culture medium with 10% FBS and then plated on a 10 cm dishes. The remaining “undigested tissue” was treated with a solution of collagenase in RPMI medium with serum containing 500 units/mL of the enzyme, incubated in a Petri dish at 37 °C for one hour. The released cells were collected, and cells were placed in a medium for culturing in the incubator. The colorectal cancer cells were cultured in Dulbecco’s Modified Eagle Medium (DMEM) medium (Gibco, Gaithersburg, MD, USA) with 10% FBS (Sigma, Co, St.Louis, MO, USA). Cells were passaged using trypsin solution (Gibco, Gaithersburg, MD, USA), once in three days at a ratio of 1:3. We used PCR-based testing with universal primers specific to the 16S rRNA region for detecting mycoplasma contaminants in cell culture [145,146,147]. Dulbecco’s Modified Eagle Medium (DMEM), Roswell Park Memorial Institute (RPMI) medium, trypsin, DMSO, puromycin and fetal bovine serum (FBS) were purchased from Sigma-Aldrich Co. (St.Louis, MO, USA).

### 4.2. Packaging of VIral Particles, Infecting Cells in Vitro, and Selecting Clones with Integrated SORE6x Reporter

We used the HEK 293T human embryonic kidney cell line to obtain viruses. Cells were transfected by the calcium phosphate method to introduce the SORE6x plasmid reporter construct (courtesy of Dr. Tang) based on the lentiviral integrating vector, as well as auxiliary plasmids to provide viral particle according to the standard protocol (https://www.epfl.ch/labs/tronolab/wp-content/uploads/2019/06/LV_production.pdf, (accessed on 19 July 2020) and our previous study [28]. Usually, infection was done twice (that is, after a day, another 10 µL of viral particles were added). Three days after the virus infection, the cells were transplanted to 10-cm culture cups (Corning Life Sciences, MA, USA) in the culture medium. The next day, the puromycin antibiotic (Sigma-Aldrich Co, St.Louis, MO, USA) was added to the medium at a concentration of 5 µg/mL. The selection followed, changing the medium with the antibiotic every two days, for 10–14 days. We selected growing colonies individually and, after treatment with trypsin, plated the cells on a six-well plate.

### 4.3. Immunofluorescence Staining of Formalin-Fixed Paraffin-Embedded Tissue Sections

We hydrated the sections gradually with graded alcohols: washed in 100% ethanol twice for 15 min each time, then in 90% ethanol twice for 15 min each time, and rinsed in deionized H_2_O for 1 min. Antigen unmasking was provided by heat treatment with 10 mM sodium citrate buffer, pH 6.0 at 95 °C for 5 min. Samples were incubated for 30 min with a blocking solution (1% horse serum in phosphate-buffered saline (PBS) and washed with three changes of PBS for 5 min each). For staining, sections were incubated with primary antibody diluted in PBS with 1% bovine serum albumin (BSA) for 60 min at room temperature or overnight at 4 °C followed by washing with three changes of PBS for 5 min each. Secondary antibodies were diluted in PBS with 1% BSA, and samples were incubated at room temperature for 60 min in a dark chamber. Immediately after washing with three changes of PBS for 5 min each, sections were covered with either an aqueous or a hard-set mounting medium. They were examined using a fluorescence microscope with appropriate filters.

### 4.4. Determining the Resistance to Cytostatics by the MTT Method

We evaluated the cell viability using a colorimetric method with MTT (Sigma-Aldrich Co., St. Louis, MO, USA). The method is based on the fact that mitochondrial oxidoreductases of the living cells restore yellow MTT to purple formazan. The amount of formazan produced correlates with the number of viable cells in the population. To determine the sensitivity of colon adenocarcinoma cells to cytostatic drugs (5-Fluorouracil, SN-38, Oxaliplatin), we seeded 50,000 cells per well of a 96-well plate in a volume of 100 µL and added 25 µL of the drug solution with a five-fold relative final concentration. The drugs were used at a concentration that allowed to determine the change in the cell line chemosensitivity (5-FU-100 ng/mL), or at a concentration for determining IC50. Cytostatic solutions were prepared in several test concentrations. For each cell line studied, we seeded the cells on six control wells–without adding the cytostatic. After incubation for two days in a cell incubator, 10 µL of the MTT solution (5 mg/mL in PBS buffer) was added to the medium followed by the incubation for 2–4 h. Then the medium was carefully removed and formazan crystals were dissolved in 100 µL of DMSO (Sigma-Aldrich Co, St.Louis, MO, USA). We measured the optical density at a wavelength of 570 nm on the Thermo Electron Multiskan EX (Invitrogen, Thermo Fisher Scientific; Waltham, MA, USA). For each experimental point, we repeated the measurement six times and calculated the standard average error. The results were presented as the percentage of cells that survived cytostatic treatment relative to the control (the number of cells that were not treated).

To verify resistance of BSC8_SORE+ cells to 5-fluorouracil in silico, we used Cancer Drug Resistance Navigator (CDRgator) (http://cdrgator.ewha.ac.kr, accessed on 15 September 2020). CDRgator is a database that unifies data on drug resistance gene signatures for about 1000 cancer cell lines and cancer cells of resistant patients (for about 30 drugs).

### 4.5. Cell Proliferation and Colony Formation Assaying

We seeded the cells into a 96-well plate at 0.5 × 10^4^ cells/well with a complete medium at 37 °C. To determine the number of live cells, we added the methylthiazolyldiphenyl-tetrazolium bromide (MTT) reagent to each well in 0, 24, 48, 72, and 96 h, respectively. The absorbance at 570 nm was detected spectrometricaly with a microplate reader (Bio-Rad Laboratories, Hercules, CA, USA). To determine clonogenic ability, we seeded the cells into a 24-well plate in the amount of 200 cells/mL in five repetitions and incubated them for 14 days. Then we stained the cell colonies with 0.01% crystal violet and counted them.

### 4.6. Soft Agar Cloning

Cells were counted, resuspended at 2 × 10^3^ cells/mL in the medium (DMEM with 10% FBS and L-glutamine) containing 0.3% weight/volume (*w/v*) agar (Bacto, Dickinson, Sparks, MD, USA) and overlaid onto a 30-mm dish containing a solidified bottom layer of 0.6% *w*/*v* agar in the same medium. After incubation for 10–15 days at 37 °C and 10% CO_2_, all dishes were stained by adding 1 mL/dish of 0.01% (*w*/*v*) crystal violet (Fronine, Taren Point, NSW, Australia), and the colonies were counted with a dissection microscope. The assaying was triplicate. The role of cultivation under hypoxic conditions was analyzed in hypoxia incubation chamber (StemcellTechnologies, Vancouver, British Columbia, Canada) with certified medical grade pre-mixed gas (1% O_2_, 5% CO_2_, 94% N_2_).

### 4.7. Wound Repair Assay

Cells were plated in 24-well plates at 10^6^ cells/well in 1 mL of the culture medium. Two days later, a wound was scratched in the adherent cell monolayers with an Eppendorf tip, and the medium was changed to DMEM supplemented with 1% FBS (Invitrogen, Thermo Fisher Scientific; Waltham, MA, USA). We examined the wells every day and took the photomicrographs using the EVOS FL Auto Imaging System (Invitrogen, Thermo Fisher Scientific; Waltham, MA, USA). Then we measured the wound width on the photomicrographs using the same well area for each measurement.

### 4.8. Migration Assay

For migration assaying, we used Transwell chambers (Corning Product; Corning, NY, USA) equipped with 8-μm-pore inserts. We plated the cells in serum-free medium on uncoated inserts and incubated them for 48 h. The volume of 600 µL of culture medium containing 20% FBS (Invitrogen, Thermo Fisher Scientific; Waltham, MA, USA) was added to the lower chamber. We removed non-invaded cells and fixed the cells attached to the bottom of the membrane with 4% paraformaldehyde, stained them with 5% crystal violet (Sigma-Aldrich Co., St. Louis, MO, USA) and counted at 200× magnification. These experiments were performed in triplicate.

### 4.9. Timelapse Microscopy and Confocal Imaging

Confocal imaging was performed on a Yokogawa CQ1 automated microscope. We used green fluorescent protein (GFP, 561 nm), 4′,6-diamidino-2-phenylindole (DAPI, 488 nm), and phase channels. The cell nuclei were stained with Hoechst 33342 (Invitrogen, Thermo Fisher Scientific; Waltham, MA, USA).

### 4.10. RNA Isolation for Transcriptome Sequencing

Total RNA was isolated from cultured cells and tissue samples (with preliminary homogenization) using the RNeasy Mini Kit (Qiagen). RNA concentration was measured on a NanoDrop 2000 spectrophotometer (Invitrogen, Thermo Fisher Scientific; Waltham, MA, USA). RNA was stored at −80 °C.

### 4.11. Transcriptome Sequencing and Analysis

NGS was done on the Illumina platform by parallel measurement of three biological samples both for BSC8_SORE+ and control cells with a read length of 150 nm. The NGS reads were processed similarly to the previous work [147]. The reads were trimmed using Trimmomatic software with default parameters [148]. The trimmed reads were mapped to the canonical nonredundant human transcriptome presented in the RefSeq database [149] using the Bowtie 2 software [150]. This aligner became a de facto standard within mapping pipelines showing a remarkable tolerance both to sequencing errors and indels [151]. We analyzed the resulting gene counts using the Limma package (implemented in the R environment) specially developed for whole transcriptome analyses of differentially expressed genes [152]. The extended version of the transcriptome analysis is presented in Supplementary Methods.

### 4.12. Gene Module Analysis

The gene module enrichment analysis was similar to the previous work [153,154,155,156]. The biological processes were taken from the GO database [157]. As a source of molecular pathways, we used the NCBI BioSystems [158]. The redundancy of this resource, which is a most complete compendium of molecular pathways from different databases, was eliminated by uniting entries with identical gene sets. The extended method is presented in Supplementary Methods.

### 4.13. Protein-Protein Interaction Network Analysis

The protein-protein interactions (PPI) were taken from the STRING database [159]. The PPI networks (PINs) were visualized using the STRING server. We analyzed the dense connected components of PINs for proteins encoded by genes differing in expression between BSC8_SORE+ cells and the control BSC8cell line, as previously [160,161,162,163,164,165,166]. Gene expression difference for genes in the network was not less than ten folds.

The up-regulated and down-regulated genes were analyzed separately.

### 4.14. Intrinsic Disorder Predisposition Analysis

We analyzed the intrinsic disorder predisposition in 219 induced and 175 inhibited proteins using a set of specialized computational tools. The global disorder propensity of target proteins (i.e., their classification as wholly ordered or wholly disordered) was evaluated by the charge hydrophathy—cumulative distribution function (CH-CDF) analysis. The CH-plot classifies query proteins as proteins with substantial amounts of extended disorder (native coils and native pre-molten globules) or proteins with compact globular conformations (native molten globules and ordered proteins) using information on their absolute mean net charge and mean hydropathy [167,168]. The CDF plot discriminates all types of disorder (native coils, native molten globules, and native pre-molten globules) from the ordered proteins [168]. Therefore, the combined CH-CDF plot (where Y-coordinate of a query protein is its distance from the boundary in the CH-plot and X-coordinate is an average distance of its CDF curve from the CDF boundary) gives an opportunity for unique assessment of intrinsic disorder in several categories, allowing predictive classification of proteins into structurally different classes [69,70,71,72]. In fact, based on their positions within the CH-CDF phase space plot, the query proteins are classified as ordered proteins (i.e., those predicted as ordered and compact by both CDF and CH; these are located within the lower-right quadrant (Q1), native molten globules or hybrid proteins containing sizable levels of order and disorder (i.e., proteins predicted to be disordered by CDF but compact by CH-plot that can be found within the lower-left quadrant (Q2), proteins with extended disorder, such as native coils and native pre-molten globules (i.e., proteins predicted to be disordered by both methods that are located within the upper-left quadrant (Q3), and proteins predicted to be disordered by CH-plot but ordered by CDF (the upper-right quadrant (Q4) [71].

Per-residue disorder predisposition of query proteins was evaluated using a set of disorder predictors from the PONDR family PONDR^®^ VLXT [169], PONDR^®^ VL3 [170], PONDR^®^ VSL2 [171] and PONDR^®^ FIT [172], as well as IUPred computational platform that allows identification of either short or long regions of intrinsic disorder, IUPred-L and IUPred-S [173,174]. The use multiple computational tools for prediction of intrinsic disorder in proteins is an accepted practice in the field. This is because different computational tools use different attributes (such as amino acid composition, hydropathy, sequence complexity, etc.) and models for to calculate a disorder predisposition score for every amino acid residue in a query protein. As a result, often, different tools generate rather different outputs. There is no accepted consensus, of which disorder predictor is the best in evaluating disorder predisposition of a query protein. In reality, since different computational tools are sensitive to different disorder-related aspects of the amino acid sequence, all of them contain some useful information.

The per-residue disorder predisposition scores are on a scale from 0 to 1, where values of 0 indicate fully-ordered residues, and values of 1 indicate fully-disordered residues. Values above the threshold of 0.5 are considered disordered residues, whereas the residues with the disorder scores between 0.25 and 0.5 are considered as highly flexible, and the residues with the disorder scores between 0.15 and 0.25 are classifies as flexible. The results of these analyses were used to classify query proteins based on their percent of predicted intrinsically disordered residues (PPIDR) and mean disorder score (MDS). Here, the accepted strategy was used to classify proteins based on their PPIDR values as highly ordered (PPIDR < 10%), moderately disordered (10% ≤ MDS < 30%), and highly disordered (PPIDR ≥ 30%) [175]. Similarly, proteins were considered as highly ordered, moderately disordered, and highly disordered if their MDS values were MDS < 0.25, 0.25 ≤ MDS < 0.5, and MDS ≥ 0.5, respectively.

### 4.15. Statistical Evaluation

RNA levels and cell viability were evaluated after three identical tests. Statistical difference in the analysis of variance was calculated using Statistica 6.0, with differences with *p* < 0.05 being considered as statistically significant. Mixed-model analysis of variance (ANOVA) or the Student’s *t*-test was used to analyze data from the luciferase reporter assays, and *p* values less than 0.05 were considered as statistically significant.

## 5. Conclusions

Achieving a significant success in the treatment of malignant tumors is impossible without effective targeting of the stem cells. It is necessary to target genes that provide unlimited proliferation and self-renewal of the stem component. The use of reporter constructs detecting the expression level of these factors as an enrichment method allows visualization and analysis of the most malignant subpopulation of tumor cells.

The use of the lentiviral reporter made it possible to isolate a high-malignant subpopulation of colon adenocarcinoma cancer cells. A comparative analysis of the transcriptome of stem-like cells allowed to characterize the main signaling pathways involved in their self-maintenance and proliferation. The results of this analysis can serve as further evidence of the high similarity of the CSC early embryonic development and functioning processes. Reversing of the stem phenotype towards terminal differentiation is a promising direction in cancer treatment. The transcriptome analysis revealed a set of genes that can serve as potential prognostic markers and therapeutic targets in colon adenocarcinoma treatment.

## Data Availability

Raw data may be provided by reasonable request.

## Supplementary Materials

The following are available online at https://www.mdpi.com/article/10.3390/ijms22094682/s1, The article is supplied by Supplementary Tables S1–S9; Supplementary Figures S1–S6, and Supplementary material and methods.

## References

[1] Bray F., Ferlay J., Soerjomataram I., Siegel R.L., Torre L.A., Jemal A. (2018). Global cancer statistics 2018: GLOBOCAN estimates of incidence and mortality worldwide for 36 cancers in 185 countries. CA Cancer J. Clin..

[2] Zacharakis M., Xynos I.D., Lazaris A., Smaro T., Kosmas C., Dokou A., Felekouras E., Antoniou E., Polyzos A., Sarantonis J. (2010). Predictors of survival in stage IV metastatic colorectal cancer. Anticancer Res..

[3] Coppedè F., Lopomo A., Spisni R., Migliore L. (2014). Genetic and epigenetic biomarkers for diagnosis, prognosis and treatment of colorectal cancer. World J. Gastroenterol..

[4] Ajani J.A., Song S., Hochster H.S., Steinberg I.B. (2015). Cancer stem cells: The promise and the potential. Semin. Oncol..

[5] Wong J.F. (2007). Probing the biology of cancer stem cells. Genet. Eng. Biotechnol. News..

[6] Dalerba P., Cho R.W., Clarke M.F. (2007). Cancer Stem Cells: Models and Concepts. Annu. Rev. Med..

[7] Tang D. (2012). Understanding cancer stem cell heterogeneity and plasticity. Cell Res..

[8] Clarke M.F., Dick J.E., Dirks P.B., Eaves C.J., Jamieson C.H.M., Jones D.L., Visvader J., Weissman I.L., Wahl G.M. (2006). Cancer stem cells—Perspectives on current status and future directions: AACR Workshop on cancer stem cells. Cancer Res..

[9] Baumann M., Krause M., Hill R. (2008). Clonogens and cancer stem cells. Nat. Rev. Cancer.

[10] Jen J., Tang Y.A., Lu Y.H., Lin C.C., Lai W.W., Wang Y.C. (2017). Oct4 transcriptionally regulates the expression of long non-coding RNAs NEAT1 and MALAT1 to promote lung cancer progression. Mol. Cancer.

[11] Shan J., Shen J., Liu L., Xia F., Xu C., Duan G., Xu Y., Ma Q., Yang Z., Zhang Q. (2012). Nanog regulates self-renewal of cancer stem cells through the insulin-like growth factor pathway in human hepatocellular carcinoma. Hepatology.

[12] Ricci-Vitiani L., Lombardi D.G., Pilozzi E., Biffoni M., Todaro M., Peschle C., de Maria R. (2007). Identification and expansion of human colon-cancer-initiating cells. Nature.

[13] O’Brien C.A., Pollett A., Gallinger S., Dick J.E. (2007). A human colon cancer cell capable of initiating tumour growth in immunodeficient mice. Nature.

[14] Todaro M., Gaggianesi M., Catalano V., Benfante A., Iovino F., Biffoni M., Apuzzo T., Sperduti I., Volpe S., Cocorullo G. (2014). CD44v6 is a marker of constitutive and reprogrammed cancer stem cells driving colon cancer metastasis. Cell Stem Cell.

[15] Yan Y., Zuo X., Wei D. (2015). Concise Review: Emerging Role of CD44 in Cancer Stem Cells: A Promising Biomarker and Therapeutic Target. Stem Cells Transl. Med..

[16] Munz M., Baeuerle P.A., Gires O. (2009). The emerging role of EpCAM in cancer and stem cell signaling. Cancer Res..

[17] Choi D., Lee H.W., Hur K.Y., Kim J.J., Park G.S., Jang S.H., Song Y.S., Jang K.S., Paik S.S. (2009). Cancer stem cell markers CD133 and CD24 correlate with invasiveness and differentiation in colorectal adenocarcinoma. World J. Gastroenterol..

[18] Tachezy M., Zander H., Gebauer F., Marx A., Kaifi J.T., Izbicki J.R., Bockhorn M. (2012). Activated leukocyte cell adhesion molecule (CD166)—Its prognostic power for colorectal cancer patients. J. Surg. Res..

[19] Corti S., Locatelli F., Papadimitriou D., Donadoni C., Salani S., del Bo R., Strazzer S., Bresolin N., Comi G.P. (2006). Identification of a Primitive Brain-Derived Neural Stem Cell Population Based on Aldehyde Dehydrogenase Activity. Stem Cells.

[20] Singh S., Arcaroli J., Chen Y., Thompson D.C., Messersmith W., Jimeno A., Vasiliou V. (2015). ALDH1B1 is crucial for colon tumorigenesis by modulating Wnt/β-catenin, Notch and PI3K/Akt signaling pathways. PLoS ONE.

[21] Huang E.H., Hynes M.J., Zhang T., Ginestier C., Dontu G., Appelman H., Fields J.Z., Wicha M.S., Boman B.M. (2009). Aldehyde dehydrogenase 1 is a marker for normal and malignant human colonic stem cells (SC) and tracks SC overpopulation during colon tumorigenesis. Cancer Res..

[22] Wu C., Alman B.A. (2008). Side population cells in human cancers. Cancer Lett..

[23] Bao S., Wu Q., McLendon R.E., Hao Y., Shi Q., Hjelmeland A.B., Dewhirst M.W., Bigner D.D., Rich J.N. (2006). Glioma stem cells promote radioresistance by preferential activation of the DNA damage response. Nature.

[24] Storms R.W., Trujillo A.P., Springer J.B., Shah L., Colvin O.M., Ludeman S.M., Smith C. (1999). Isolation of primitive human hematopoietic progenitors on the basis of aldehyde dehydrogenase activity. Proc. Natl. Acad. Sci. USA.

[25] Shmelkov S.V., Butler J.M., Hooper A.T., Hormigo A., Kushner J., Milde T., Clair R.S., Baljevic M., White I., Jin D.K. (2008). CD133 expression is not restricted to stem cells, and both CD133^+^ and CD133^−^ metastatic colon cancer cells initiate tumors. J. Clin. Investig..

[26] Masters J.R., Foley C.L., Bisson I., Ahmed A. (2003). Cancer stem cells. BJU Int..

[27] Saygin C., Samour M., Chumakova A., Jarrar A., Lathia J.D., Reizes O. (2016). Reporter systems to study cancer stem cells. Methods Mol. Biol..

[28] Tang B., Raviv A., Esposito D., Flanders K.C., Daniel C., Nghiem B.T., Garfield S., Lim L., Mannan P., Robles A.I. (2015). A flexible reporter system for direct observation and isolation of cancer stem cells. Stem Cell Rep..

[29] Wiechert A., Saygin C., Thiagarajan P.S., Rao V.S., Hale J.S., Gupta N., Hitomi M., Nagaraj A.B., DiFeo A., Lathia J.D. (2016). Cisplatin induces stemness in ovarian cancer. Oncotarget.

[30] Kurtova A.V., Xiao J., Mo Q., Pazhanisamy S.K., Krasnow R., Lerner S.P., Chen F., Roh T.T., Lay E., Ho P.L. (2015). Blocking PGE2-induced tumour repopulation abrogates bladder cancer chemoresistance. Nature.

[31] Cherepanova O.A., Gomez D., Shankman L.S., Swiatlowska P., Williams J., Sarmento O.F., Alencar G.F., Hess D.L., Bevard M.H., Greene E.S. (2016). Activation of the pluripotency factor OCT4 in smooth muscle cells is atheroprotective. Nat. Med..

[32] Liu C.W., Li C.H., Peng Y.J., Cheng Y.W., Chen H.W., Liao P.L., Kang J.J., Yeng M.H. (2014). Snail regulates Nanog status during the epithelial-mesenchymal transition via the Smad1/Akt/GSK3β signaling pathway in non-small-cell lung cancer. Oncotarget.

[33] Liu A.Y., Cai Y., Mao Y., Lin Y., Zheng H., Wu T., Huang Y., Fang X., Lin S., Feng Q. (2014). Twist2 promotes self-renewal of liver cancer stem-like cells by regulating CD24. Carcinogenesis.

[34] Li P., Yang R., Gao W.Q. (2014). Contributions of epithelial-mesenchymal transition and cancer stem cells to the development of castration resistance of prostate cancer. Mol. Cancer.

[35] Chaffer C.L., Marjanovic N.D., Lee T., Bell G., Kleer C.G., Reinhardt F., D’Alessio A.C., Young R.A., Weinberg R.A. (2013). XPoised chromatin at the ZEB1 promoter enables breast cancer cell plasticity and enhances tumorigenicity. Cell.

[36] Martin A., Cano A. (2010). Tumorigenesis: Twist1 links EMT to self-renewal. Nat. Cell Biol..

[37] Singh A., Settleman J. (2010). EMT, cancer stem cells and drug resistance: An emerging axis of evil in the war on cancer. Oncogene.

[38] Meng S., Chanda P., Thandavarayan R.A., Cooke J.P. (2018). Transflammation: How Innate Immune Activation and Free Radicals Drive Nuclear Reprogramming. Antioxid. Redox Signal..

[39] Choi J., Baek K.H. (2018). Cellular functions of stem cell factors mediated by the ubiquitin—Proteasome system. Cell. Mol. Life Sci..

[40] Boutet A. (2017). The evolution of asymmetric photosensitive structures in metazoans and the Nodal connection. Mech. Dev..

[41] Machida K. (2013). Tumor-initiating stem-like cells and drug resistance: Carcinogenesis through Toll-like receptors, environmental factors, and virus. Drug Deliv. Transl. Res..

[42] Harbuzariu A., Gonzalez-Perez R.R. (2018). Leptin-Notch axis impairs 5-fluorouracil effects on pancreatic cancer. Oncotarget.

[43] Davis J.E., Kirk J., Ji Y., Tang D.G. (2019). Tumor Dormancy and Slow-Cycling Cancer Cells. Advances in Experimental Medicine and Biology.

[44] Eggenberger J., Blanco-Melo D., Panis M., Brennand K.J., Ten Oever B.R. (2019). Type I interferon response impairs differentiation potential of pluripotent stem cells. Proc. Natl. Acad. Sci. USA.

[45] Jang S.K., Yoon B.H., Kang S.M., Yoon Y.G., Kim S.Y., Kim W. (2019). Cdrgator: An integrative navigator of cancer drug resistance gene signatures. Mol. Cells.

[46] Dunker A.K., Lawson J.D., Brown C.J., Williams R.M., Romero P., Oh J.S., Oldfield C.J., Campen A.M., Ratliff C.M., Hipps K.W. (2001). Intrinsically disordered protein. J. Mol. Graph. Model..

[47] Uversky V.N., Dunker A.K. (2010). Understanding protein non-folding. Biochim. Biophys. Acta.

[48] Uversky V.N. (2013). Unusual biophysics of intrinsically disordered proteins. Biochim. Biophys. Acta.

[49] Uversky V.N. (2013). A decade and a half of protein intrinsic disorder: Biology still waits for physics. Protein Sci..

[50] Uversky V.N. (2013). Intrinsic disorder-based protein interactions and their modulators. Curr. Pharm. Des..

[51] Oldfield C.J., Dunker A.K. (2014). Intrinsically disordered proteins and intrinsically disordered protein regions. Annu. Rev. Biochem..

[52] Uversky V.N. (2011). Multitude of binding modes attainable by intrinsically disordered proteins: A portrait gallery of disorder-based complexes. Chem. Soc. Rev..

[53] Uversky V.N. (2015). The multifaceted roles of intrinsic disorder in protein complexes. FEBS Lett..

[54] Mammen M., Choi S.K., Whitesides G.M. (1998). Polyvalent interactions in biological systems: Implications for design and use of multivalent ligands and inhibitors. Angew. Chem. Int. Ed..

[55] Schulz G.E., Balaban M. (1979). Nucleotide Binding Proteins. Molecular Mechanism of Biological Recognition.

[56] Wright P.E., Dyson H.J. (2009). Linking folding and binding. Curr. Opin. Struct. Biol..

[57] Dyson H.J., Wright P.E. (2002). Coupling of folding and binding for unstructured proteins. Curr. Opin. Struct. Biol..

[58] Dyson H.J., Wright P.E. (2005). Intrinsically unstructured proteins and their functions. Nat. Rev. Mol. Cell Biol..

[59] van der Lee R., Buljan M., Lang B., Weatheritt R.J., Daughdrill G.W., Dunker A.K., Fuxreiter M., Gough J., Gsponer J., Jones D.T. (2014). Classification of intrinsically disordered regions and proteins. Chem. Rev..

[60] Meng J., Yang J.Y., Yang M.Q., Uversky V.N., Dunker A.K. (2008). Flexible nets: Disorder and induced fit in the associations of p53 and 14-3-3 with their partners. BMC Genom..

[61] Dunker A.K., Cortese M.S., Romero P., Iakoucheva L.M., Uversky V.N. (2005). Flexible nets: The roles of intrinsic disorder in protein interaction networks. FEBS J..

[62] Uversky V.N. (2019). Intrinsically disordered proteins and their “mysterious” (meta)physics. Front. Phys..

[63] Uversky V.N. (2019). Protein intrinsic disorder and structure-function continuum. Prog. Mol. Biol. Transl. Sci..

[64] Patil A., Nakamura H. (2006). Disordered domains and high surface charge confer hubs with the ability to interact with multiple proteins in interaction networks. FEBS Lett..

[65] Ekman D., Light S., Bjorklund A.K., Elofsson A. (2006). What properties characterize the hub proteins of the protein-protein interaction network of Saccharomyces cerevisiae?. Genome Biol..

[66] Haynes C., Oldfield C.J., Ji F., Klitgord N., Cusick M.E., Radivojac P., Uversky V.N., Vidal M., Iakoucheva L.M. (2006). Intrinsic disorder is a common feature of hub proteins from four eukaryotic interactomes. PLoS Comput. Biol..

[67] Dosztanyi Z., Chen J., Dunker A.K., Simon I., Tompa P. (2006). Disorder and sequence repeats in hub proteins and their implications for network evolution. J. Proteome Res..

[68] Dash D. (2007). Intrinsic disorder in yeast transcriptional regulatory network. Proteins.

[69] Singh G.P., Ganapathi M., Dash D. (2007). Role of intrinsic disorder in transient interactions of hub proteins. Proteins.

[70] Xue B., Oldfield C.J., Dunker A.K., Uversky V.N. (2009). CDF it all: Consensus prediction of intrinsically disordered proteins based on various cumulative distribution functions. FEBS Lett..

[71] Huang F., Oldfield C.J., Xue B., Hsu W.L., Meng J., Liu X., Shen L., Romero P., Uversky V.N., Dunker A. (2014). Improving protein order-disorder classification using charge-hydropathy plots. BMC Bioinform..

[72] Mohan A., Sullivan W.J., Radivojac P., Dunker A.K., Uversky V.N. (2008). Intrinsic disorder in pathogenic and non-pathogenic microbes: Discovering and analyzing the unfoldomes of early-branching eukaryotes. Mol. BioSyst..

[73] Huang F., Oldfield C., Meng J., Hsu W.L., Xue B., Uversky V.N., Romero P., Dunker A.K. (2012). Subclassifying disordered proteins by the CH-CDF plot method. Pas. Symp. Biocomputing.

[74] Santoro A., Vlachou T., Carminati M., Pelicci P.G., Mapelli M. (2016). Molecular mechanisms of asymmetric divisions in mammary stem cells. EMBO Rep..

[75] Costa G., Harrington K.I., Lovegrove H.E., Page D.J., Chakravartula S., Bentley K., Herbert S.P. (2016). Asymmetric division coordinates collective cell migration in angiogenesis. Nat. Cell Biol..

[76] Pece S., Tosoni D., Confalonieri S., Mazzarol G., Vecchi M., Ronzoni S., Bernard L., Viale G., Pelicci P.G., Di Fiore P.P. (2010). Biological and molecular heterogeneity of breast cancers correlates with their cancer stem cell content. Cell.

[77] Franken N.A.P., Rodermond H.M., Stap J., Haveman J., van Bree C. (2006). Clonogenic assay of cells in vitro. Nat. Protoc..

[78] Aiken C., Werbowetski-Ogilvie T. (2013). Animal Models of Cancer Stem Cells: What are They Really Telling Us?. Curr. Pathobiol. Rep..

[79] Fujino S., Miyoshi N. (2019). OCT4 gene expression in primary colorectal cancer promotes liver metastasis. Stem Cells Int..

[80] Cai W., Wang Z., Wei C., Wu M., Zheng W., Zhang H., Liu C., Liu L. (2019). Prognostic evaluation of NANOG and OCT4 expression for posttransplantation hepatocellular carcinoma recurrence. J. Cell. Biochem..

[81] Basati G., Mohammadpour H., Emami Razavi A. (2020). Association of High Expression Levels of SOX2, NANOG, and OCT4 in Gastric Cancer Tumor Tissues with Progression and Poor Prognosis. J. Gastrointest. Cancer.

[82] Mohiuddin I.S., Wei S.J., Kang M.H. (2020). Role of OCT4 in cancer stem-like cells and chemotherapy resistance. Biochim. Biophys. Acta Mol. Basis Dis..

[83] Li W., Zhou Y., Zhang X., Yang Y., Dan S., Su T., She S., Dong W., Zhao Q., Jia J. (2017). Dual inhibiting OCT4 and AKT potently suppresses the propagation of human cancer cells. Sci. Rep..

[84] Forghanifard M.M., Moghbeli M., Raeisossadati R., Tavassoli A., Mallak A.J., Boroumand-Noughabi S., Abbaszadegan M.R. (2013). Role of SALL4 in the progression and metastasis of colorectal cancer. J. Biomed. Sci..

[85] Nicolè L., Sanavia T., Veronese N., Cappellesso R., Luchini C., Dabrilli P., Fassina A. (2017). Oncofetal gene SALL4 and prognosis in cancer: A systematic review with meta-analysis. Oncotarget.

[86] Tatetsu H., Kong N.R., Chong G., Amabile G., Tenen D.G., Chai L. (2016). SALL4, the missing link between stem cells, development and cancer. Gene.

[87] Wang J.H., Wei W., Xu J., Guo Z.X., Xiao C.Z., Zhang Y.F., Jian P.E., Wu X.L., Shi M., Guo R.P. (2015). Elevated expression of Cripto-1 correlates with poor prognosis in hepatocellular carcinoma. Oncotarget.

[88] Duan W., Li R., Ma J., Lei J., Xu Q., Jiang Z., Nan L., Li X., Wang Z., Huo X. (2015). Overexpression of Nodal induces a metastatic phenotype in pancreatic cancer cells via the Smad2/3 pathway. Oncotarget.

[89] Huang C., Chen W., Wang X., Zhao J., Li Q., Fu Z. (2015). Cripto-1 Promotes the Epithelial-Mesenchymal Transition in Esophageal Squamous Cell Carcinoma Cells. Evid. Based Complement. Altern. Med..

[90] Klauzinska M., Castro N.P., Rangel M.C., Spike B.T., Gray P.C., Bertolette D., Cuttitta F., Salomon D. (2014). The multifaceted role of the embryonic gene Cripto-1 in cancer, stem cells and epithelial-mesenchymal transition. Semin. Cancer Biol..

[91] Lonardo E., Hermann P.C., Mueller M.-T., Huber S., Balic A., Miranda-Lorenzo I., Zagorac S., Alcala S., Rodriguez-Arabaolaza I., Ramirez J.C. (2011). Nodal/activin signaling drives self-renewal and tumorigenicity of pancreatic cancer stem cells and provides a target for combined drug therapy. Cell Stem Cell.

[92] Wilson B.J., Schatton T., Zhan Q., Gasser M., Jie M., Saab K.R., Schanche R., Waaga-Gasser A.M., Gold J.S., Huang Q. (2011). ABCB5 identifies a therapy-refractory tumor cell population in colorectal cancer patients. Cancer Res..

[93] Kugimiya N., Nishimoto A., Hosoyama T., Ueno K., Enoki T., Li T.S., Hamano K. (2015). The c-MYC-ABCB5 axis plays a pivotal role in 5-fluorouracil resistance in human colon cancer cells. J. Cell. Mol. Med..

[94] Grimm M., Krimmel M., Polligkeit J., Alexander D., Munz A., Kluba S., Keutel C., Hoffmann J., Reinert S., Hoefert S. (2012). ABCB5 expression and cancer stem cell hypothesis in oral squamous cell carcinoma. Eur. J. Cancer.

[95] Guo Q., Grimmig T., Gonzalez G., Giobbie-Hurder A., Berg G., Carr N., Wilson B.J., Banerjee P., Ma J., Gold J.S. (2018). ATP-binding cassette member B5 (ABCB5) promotes tumor cell invasiveness in human colorectal cancer. J. Biol. Chem..

[96] Ferdinande L., Decaestecker C., Verset L., Mathieu A., Moles Lopez X., Negulescu A.M., van Maerken T., Salmon I., Cuvelier C.A., Demetter P. (2012). Clinicopathological significance of indoleamine 2,3-dioxygenase 1 expression in colorectal cancer. Br. J. Cancer.

[97] Uyttenhove C., Pilotte L., Théate I., Stroobant V., Colau D., Parmentier N., Boon T., van den Eynde B.J. (2003). Evidence for a tumoral immune resistance mechanism based on tryptophan degradation by indoleamine 2,3-dioxygenase. Nat. Med..

[98] Moffett J.R., Namboodiri M.A. (2003). Tryptophan and the immune response. Immunol. Cell Biol..

[99] Fallarino F., Grohmann U., You S., McGrath B.C., Cavener D.R., Vacca C., Orabona C., Bianchi R., Belladonna M.L., Volpi C. (2006). The Combined Effects of Tryptophan Starvation and Tryptophan Catabolites Down-Regulate T Cell Receptor ζ-Chain and Induce a Regulatory Phenotype in Naive T Cells. J. Immunol..

[100] Lindeman G.J., Visvader J.E. (2010). Insights into the cell of origin in breast cancer and breast cancer stem cells. Asia Pac. J. Clin. Oncol..

[101] Li S., Kennedy M., Payne S., Kennedy K., Seewaldt V.L., Pizzo S.V., Bachelder R.E. (2014). Model of tumor dormancy/recurrence after short-term chemotherapy. PLoS ONE.

[102] Dembinski J.L., Krauss S. (2010). A distinct slow-cycling cancer stem-like subpopulation of pancreatic adenocarcinoma cells is maintained in Vivo. Cancers.

[103] Atkins R.J., Stylli S.S., Kurganovs N., Mangiola S., Nowell C.J., Ware T.M., Corcoran N.M., Brown D.V., Kaye A.H., Morokoff A. (2019). Cell quiescence correlates with enhanced glioblastoma cell invasion and cytotoxic resistance. Exp. Cell Res..

[104] Ohlsson L., Hammarström M.L., Lindmark G., Hammarström S., Sitohy B. (2016). Ectopic expression of the chemokine CXCL17 in colon cancer cells. Br. J. Cancer.

[105] Sjöberg E., Meyrath M., Milde L., Herrera M., Lövrot J., Hägerstrand D., Frings O., Bartish M., Rolny C., Sonnhammer E. (2019). A novel ACKR2-Dependent role of fibroblast-derived CXCL14 in epithelial-to-mesenchymal transition and metastasis of breast cancer. Clin. Cancer Res..

[106] Yang X.L., Liu K.Y., Lin F.J., Shi H.M., Ou Z.L. (2017). CCL28 promotes breast cancer growth and metastasis through MAPK-mediated cellular anti-apoptosis and pro-metastasis. Oncol. Rep..

[107] Yang G., Rosen D., Liu G., Yang F., Guo X., Xiao X., Xue F., Mercado-Uribe I., Huang J., Lin S.H. (2010). CXCR2 promotes ovarian cancer growth through dysregulated cell cycle, diminished apoptosis, and enhanced angiogenesis. Clin. Cancer Res..

[108] Cheng W.L., Wang C.S., Huang Y.H., Tsai M.M., Liang Y., Lin K.H. (2011). Overexpression of CXCL1 and its receptor CXCR2 promote tumor invasion in gastric cancer. Ann. Oncol..

[109] Wang Z., Liu H., Shen Z., Wang X., Zhang H., Qin J., Xu J., Sun Y., Qin X. (2015). The prognostic value of CXC-chemokine receptor 2 (CXCR2) in gastric cancer patients. BMC Cancer.

[110] Zhao J., Ou B., Feng H., Wang P., Yin S., Zhu C., Wang S., Chen C., Zheng M., Zong Y. (2017). Overexpression of CXCR2 predicts poor prognosis in patients with colorectal cancer. Oncotarget.

[111] Varney M.L., Singh S., Li A., Mayer-Ezell R., Bond R., Singh R.K. (2011). Small molecule antagonists for CXCR2 and CXCR1 inhibit human colon cancer liver metastases. Cancer Lett..

[112] Augustin H.G., Young Koh G., Thurston G., Alitalo K. (2009). Control of vascular morphogenesis and homeostasis through the angiopoietin—Tie system. Nat. Rev. Mol. Cell Biol..

[113] Gavalas N.G., Liontos M., Trachana S.P., Bagratuni T., Arapinis C., Liacos C., Dimopoulos M.A., Bamias A. (2013). Angiogenesis-related pathways in the pathogenesis of ovarian cancer. Int. J. Mol. Sci..

[114] Sfiligoi C., de Luca A., Cascone I., Sorbello V., Fuso L., Ponzone R., Biglia N., Audero E., Arisio R., Bussolino F. (2003). Angiopoietin-2 expression in breast cancer correlates with lymph node invasion and short survival. Int. J. Cancer.

[115] Mitsuhashi N., Shimizu H., Ohtsuka M., Wakabayashi Y., Ito H., Kimura F., Yoshidome H., Kato A., Nukui Y., Miyazaki M. (2003). Angiopoietins and Tie-2 expression in angiogenesis and proliferation of human hepatocellular carcinoma. Hepatology.

[116] Ramanathan R., Olex A.L., Dozmorov M., Bear H.D., Fernandez L.J., Takabe K. (2017). Angiopoietin pathway gene expression associated with poor breast cancer survival. Breast Cancer Res. Treat..

[117] Huang H., Bhat A., Woodnutt G., Lappe R. (2010). Targeting the ANGPT-TIE2 pathway in malignancy. Nat. Rev. Cancer.

[118] Dai J., Wan S., Zhou F., Myers R.E., Guo X., Li B., Fu X., Palazzo J.P., Dou K., Yang H. (2012). Genetic polymorphism in a vegf-independent angiogenesis gene angpt1 and overall survival of colorectal cancer patients after surgical resection. PLoS ONE.

[119] Cortese M.S., Uversky V.N., Dunker A. (2008). Intrinsic disorder in scaffold proteins: Getting more from less. Prog. Biophys. Mol. Biol..

[120] Dunker A.K., Brown C.J., Lawson J.D., Iakoucheva L.M., Obradovic Z. (2002). Intrinsic disorder and protein function. Biochemistry.

[121] Dunker A.K., Obradovic Z. (2001). The protein trinity-linking function and disorder. Nat. Biotechnol..

[122] Dunker A.K., Silman I., Uversky V.N., Sussman J.L. (2008). Function and structure of inherently disordered proteins. Curr. Opin. Struct. Biol..

[123] Dunker A.K., Uversky V.N. (2008). Signal transduction via unstructured protein conduits. Nat. Chem. Biol..

[124] Iakoucheva L.M., Brown C.J., Lawson J.D., Obradovic Z., Dunker A.K. (2002). Intrinsic disorder in cell-signaling and cancer-associated proteins. J. Mol. Biol..

[125] Radivojac P., Iakoucheva L.M., Oldfield C.J., Obradovic Z., Uversky V.N., Dunker A.K. (2007). Intrinsic Disorder and Functional Proteomics. Biophys. J..

[126] Tompa P. (2002). Intrinsically unstructured proteins. Trends Biochem. Sci..

[127] Uversky V.N. (2002). Natively unfolded proteins: A point where biology waits for physics. Protein Sci..

[128] Uversky V.N. (2002). What does it mean to be natively unfolded?. Eur. J. Biochem..

[129] Uversky V.N. (2010). The mysterious unfoldome: Structureless, underappreciated, yet vital part of any given proteome. J. Biomed. Biotechnol..

[130] Uversky V.N., Oldfield C.J., Dunker A.K. (2005). Showing your ID: Intrinsic disorder as an ID for recognition, regulation and cell signaling. J. Mol. Recognit..

[131] Vucetic S., Xie H., Iakoucheva L.M., Oldfield C.J., Dunker A.K., Obradovic Z., Uversky V.N. (2007). Functional anthology of intrinsic disorder. 2. Cellular components, domains, technical terms, developmental processes, and coding sequence diversities correlated with long disordered regions. J. Proteome Res..

[132] Xie H., Vucetic S., Iakoucheva L.M., Oldfield C.J., Dunker A.K., Obradovic Z., Uversky V.N. (2007). Functional anthology of intrinsic disorder. 3. Ligands, post-translational modifications, and diseases associated with intrinsically disordered proteins. J. Proteome Res..

[133] Xie H., Vucetic S., Iakoucheva L.M., Oldfield C.J., Dunker A.K., Uversky V.N., Obradovic Z. (2007). Functional anthology of intrinsic disorder. 1. Biological processes and functions of proteins with long disordered regions. J. Proteome Res..

[134] Uversky V.N., Oldfield C.J., Dunker A.K. (2008). Intrinsically disordered proteins in human diseases: Introducing the D^2^ concept. Annu. Rev. Biophys..

[135] Uversky V.N., Dave V., Iakoucheva L.M., Malaney P., Metallo S.J., Pathak R.R., Joerger A.C. (2014). Pathological unfoldomics of uncontrolled chaos: Intrinsically disordered proteins and human diseases. Chem. Rev..

[136] Uversky V.N. (2008). Amyloidogenesis of natively unfolded proteins. Curr. Alzheimer Res..

[137] Cheng Y., LeGall T., Oldfield C.J., Dunker A.K., Uversky V.N. (2006). Abundance of intrinsic disorder in protein associated with cardiovascular disease. Biochemistry.

[138] Du Z., Uversky V.N. (2017). A Comprehensive Survey of the Roles of Highly Disordered Proteins in Type 2 Diabetes. Int. J. Mol. Sci..

[139] Uversky V.N. (2014). The triple power of D^3^: Protein intrinsic disorder in degenerative diseases. Front. Biosci..

[140] Uversky V.N. (2009). Intrinsic disorder in proteins associated with neurodegenerative diseases. Front. Biosci..

[141] Uversky V.N. (2014). Wrecked regulation of intrinsically disordered proteins in diseases: Pathogenicity of deregulated regulators. Front. Mol. Biosci..

[142] Uversky V.N., Oldfield C.J., Midic U., Xie H., Xue B., Vucetic S., Iakoucheva L.M., Obradovic Z., Dunker A.K. (2009). Unfoldomics of human diseases: Linking protein intrinsic disorder with diseases. BMC Genom..

[143] Xue B., Oldfield C.J., Van Y.-Y., Dunker A.K., Uversky V.N. (2012). Protein intrinsic disorder and induced pluripotent stem cells. Mol. BioSyst..

[144] Yu C.S., Huang A.C., Lai K.C., Huang Y.P., Lin M.W., Yang J.S., Chung J.G. (2012). Diallyl trisulfide induces apoptosis in human primary colorectal cancer cells. Oncol. Rep..

[145] Koshkin S., Danilova A., Raskin G., Petrov N., Bajenova O., O’Brien S.J., Tomilin A., Tolkunova E. (2016). Primary cultures of human colon cancer as a model to study cancer stem cells. Tumour Biol..

[146] Young L., Sung J., Masters J.R. (2010). Detection of mycoplasma in cell cultures. Nat. Protoc..

[147] Bajenova O., Chaika N., Tolkunova E., Davydov-Sinitsyn A., Gapon S., Thomas P., O’Brien S. (2014). Carcinoembryonic antigen promotes colorectal cancer progression by targeting adherens junction complexes. Exp. Cell Res..

[148] Vinogradov A.E., Shilina M.A., Anatskaya O.V., Alekseenko L.L., Fridlyanskaya I.I., Krasnenko A., Kim A., Korostin D., Ilynsky V., Elmuratov A. (2017). Molecular genetic analysis of human endometrial mesenchymal stem cells that survived sublethal heat shock. Stem Cells Int..

[149] Bolger A.M., Lohse M., Usadel B. (2014). Trimmomatic: A flexible trimmer for Illumina sequence data. Bioinformatics.

[150] Agarwala R., Barrett T., Beck J., Benson D.A., Bollin C., Bolton E., Bourexis D., Brister J.R., Bryant S.H., Canese K. (2018). Database resources of the National Center for Biotechnology Information. Nucleic Acids Res..

[151] Langmead B., Salzberg S.L. (2012). Fast gapped-read alignment with Bowtie 2. Nat. Methods.

[152] Lindner R., Friedel C.C. (2012). A Comprehensive Evaluation of Alignment Algorithms in the Context of RNA-Seq. PLoS ONE.

[153] Ritchie M.E., Phipson B., Wu D., Hu Y., Law C.W., Shi W., Smyth G.K. (2015). Limma powers differential expression analyses for RNA-sequencing and microarray studies. Nucleic Acids Res..

[154] Vinogradov A.E., Anatskaya O.V. (2019). Gene Golden Age paradox and its partial solution. Genomics.

[155] Anatskaya O.V., Vinogradov A.E., Vainshelbaum N.M., Giuliani A., Erenpreisa J. (2020). Phylostratic Shift of Whole-Genome Duplications in Normal Mammalian Tissues towards Unicellularity Is Driven by Developmental Bivalent Genes and Reveals a Link to Cancer. Int. J. Mol. Sci..

[156] Vinogradov A.E., Anatskaya O.V. (2020). Cell-cycle dependence of transcriptome gene modules: Comparison of regression lines. FEBS J..

[157] Carbon S., Douglass E., Dunn N., Good B., Harris N.L., Lewis S.E., Mungall C.J., Basu S., Chisholm R.L., Dodson R.J. (2019). The Gene Ontology Resource: 20 years and still GOing strong. Nucleic Acids Res..

[158] Sayers E.W., Beck J., Brister J.R., Bolton E.E., Canese K., Comeau D.C., Funk K., Ketter A., Kim S., Kimchi A. (2020). Database resources of the National Center for Biotechnology Information. Nucleic Acids Res..

[159] Szklarczyk D., Franceschini A., Wyder S., Forslund K., Heller D., Huerta-Cepas J., Simonovic M., Roth A., Santos A., Tsafou K.P. (2015). STRING v10: Protein—Protein interaction networks, integrated over the tree of life. Nucleic Acids Res..

[160] Vazquez-Martin A., Anatskaya O.V., Giuliani A., Erenpreisa J., Huang S., Salmina K., Inashkina I., Huna A., Nikolsky N.N., Vinogradov A.E. (2016). Somatic polyploidy is associated with the upregulation of c-MYC interacting genes and EMT-like signature. Oncotarget.

[161] Erenpreisa J., Salmina K., Anatskaya O., Cragg M.S. (2020). Paradoxes of cancer: Survival at the brink. Seminars in Cancer Biology.

[162] Shilina M.A., Grinchuk T.M., Anatskaya O.V., Vinogradov A.E., Alekseenko L.L., Elmuratov A.U., Nikolsky N.N. (2018). Cytogenetic and Transcriptomic Analysis of Human Endometrial MSC Retaining Proliferative Activity after Sublethal Heat Shock. Cells.

[163] Vinogradov A.E., Anatskaya O.V. (2020). Systemic evolutionary changes in mammalian gene expression. Biosystems.

[164] Vinogradov A.E., Anatskaya O.V. (2019). Evolutionary framework of the human interactome: Unicellular and multicellular giant clusters. Biosystems.

[165] Vinogradov A.E., Anatskaya O.V. (2017). DNA helix: The importance of being AT-rich. Mamm. Genome.

[166] Anatskaya O.V., Vinogradov A.E. (2010). Somatic polyploidy promotes cell function under stress and energy depletion: Evidence from tissue-specific mammal transcriptome. Funct. Integr. Genom..

[167] Uversky V.N., Gillespie J.R., Fink A.L. (2000). Why are “natively unfolded” proteins unstructured under physiologic conditions?. Proteins.

[168] Oldfield C.J., Cheng Y., Cortese M.S., Brown C.J., Uversky V.N., Dunker A.K. (2005). Comparing and combining predictors of mostly disordered proteins. Biochemistry.

[169] Romero P., Obradovic Z., Li X., Garner E.C., Brown C.J., Dunker A.K. (2001). Sequence complexity of disordered protein. Proteins.

[170] Peng K., Radivojac P., Vucetic S., Dunker A.K., Obradovic Z. (2006). Length-dependent prediction of protein intrinsic disorder. BMC Bioinform..

[171] Peng K., Vucetic S., Radivojac P., Brown C.J., Dunker A.K., Obradovic Z. (2005). Optimizing long intrinsic disorder predictors with protein evolutionary information. J. Bioinform. Comput. Biol..

[172] Xue B., Dunbrack R.L., Williams R.W., Dunker A.K., Uversky V.N. (2010). PONDR-FIT: A meta-predictor of intrinsically disordered amino acids. Biochim. Biophys. Acta.

[173] Dosztányi Z., Csizmok V., Tompa P., Simon I. (2005). IUPred: Web server for the prediction of intrinsically unstructured regions of proteins based on estimated energy content. Bioinformatics.

[174] Dosztanyi Z., Csizmok V., Tompa P., Simon I. (2005). The pairwise energy content estimated from amino acid composition discriminates between folded and intrinsically unstructured proteins. J. Mol. Biol..

[175] Rajagopalan K., Mooney S.M., Parekh N., Getzenberg R.H., Kulkarni P. (2011). A majority of the cancer/testis antigens are intrinsically disordered proteins. J. Cell. Biochem..

