# Supplementary material for: Isolation and Characterization of Human Colon Adenocarcinoma Stem-Like Cells Based on the Endogenous Expression of the Stem Markers
Source: Int J Mol Sci. 2021 Apr 28;22(9):4682. doi: 10.3390/ijms22094682 (PMC8124683; doi:10.3390/ijms22094682)
Supplement: Supplementary file 1 [file ijms-22-04682-s001.zip › Supplementary figures.pdf]

Supplementary Figures S1-S5 with extended legends

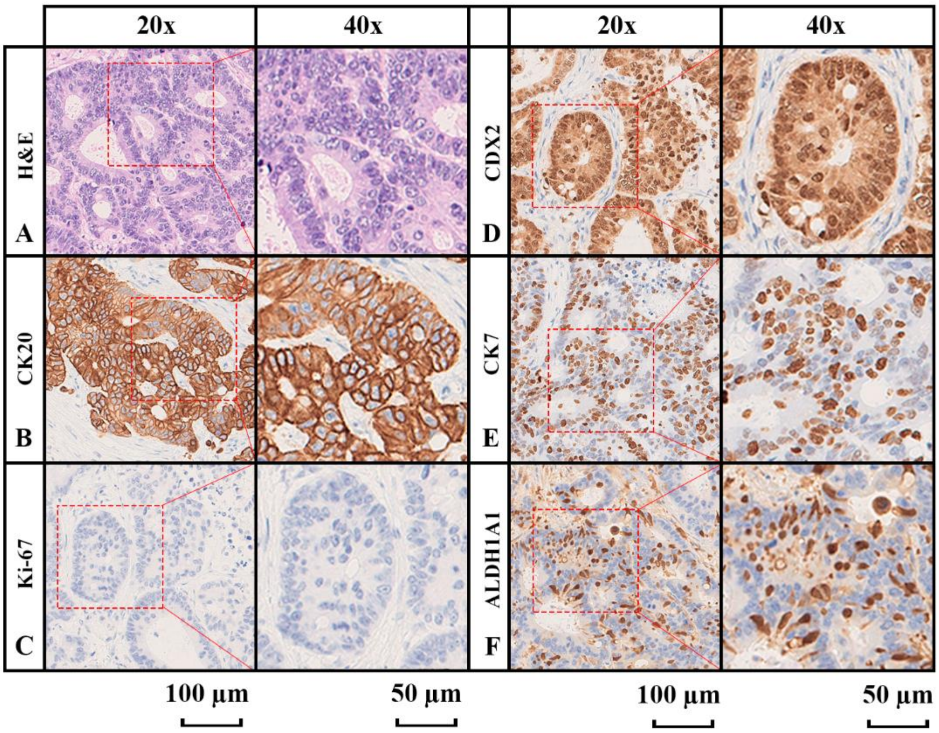

Suppl. Fig. S1. HE and IHC staining of colon cancer sample - source of primary cuture BSC8. **A-** H&E- stainig. The histostructure corresponds to a moderately differentiated colon adenocarcinoma. More than 50% of the cells form tumor glands. **B** - anti-CK20 staining. 80% cancer cells have positive membnouse staining. **C** – Anti-Ki-67 stainig. 40% of cancer cells prolirativelу active. **D**-anti-CDX2 staining. All cancer cells have strong nuclear CDX2 expression. **E** - Negative anti-CK7 staining. **F**. Anti- ALDH1A1 staining- 35% of cells ALDH1A1. Magnification - 20 x, 40x. Scale bar - 100 and 50 μm.

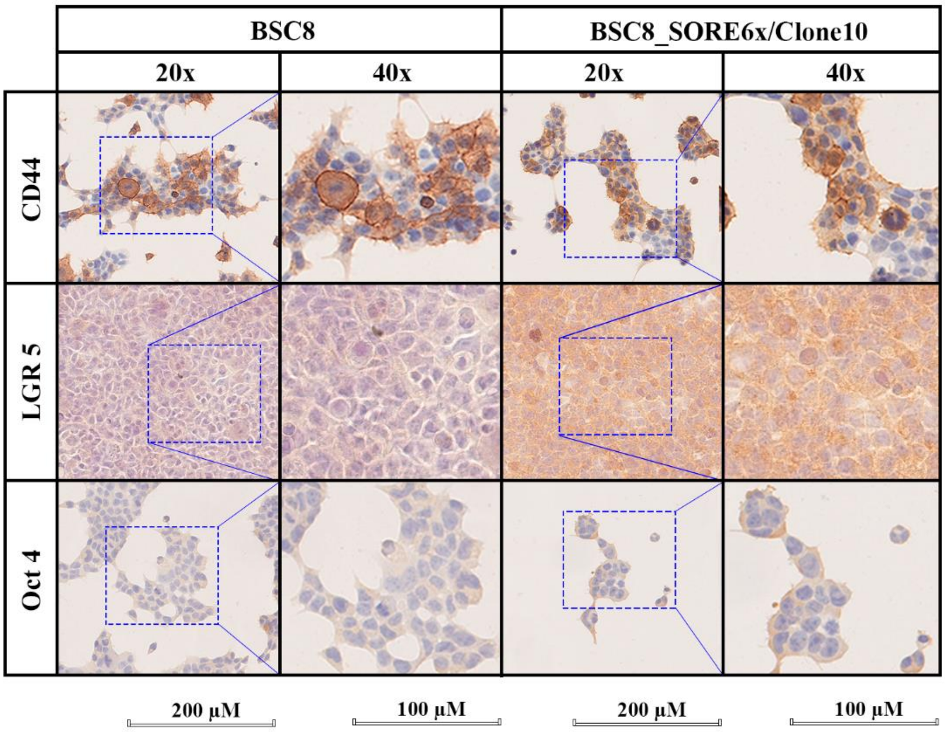

Suppl. Fig. S2. Expression of stem cell markers in BSC8 and BSC8_SORE+/Clone10 cells. A. Anti-CD44 staining of cancer cells in culture. Arrows indicate cells with membrane and cytoplasmic staining. In both cell lines, an equal proportion of cells express CD44 marker. **B.** Anti-Lgr-5 staining of cancer cells in culture. BSC8_SORE+/Clone10 cells express a higher level of LGR-5. C. Anti-Oct4 immunostaining of cancer cells in culture. There is a weak cytoplasmic expression of Oct4 in cancer cells. Magnification is 20x and 40x.

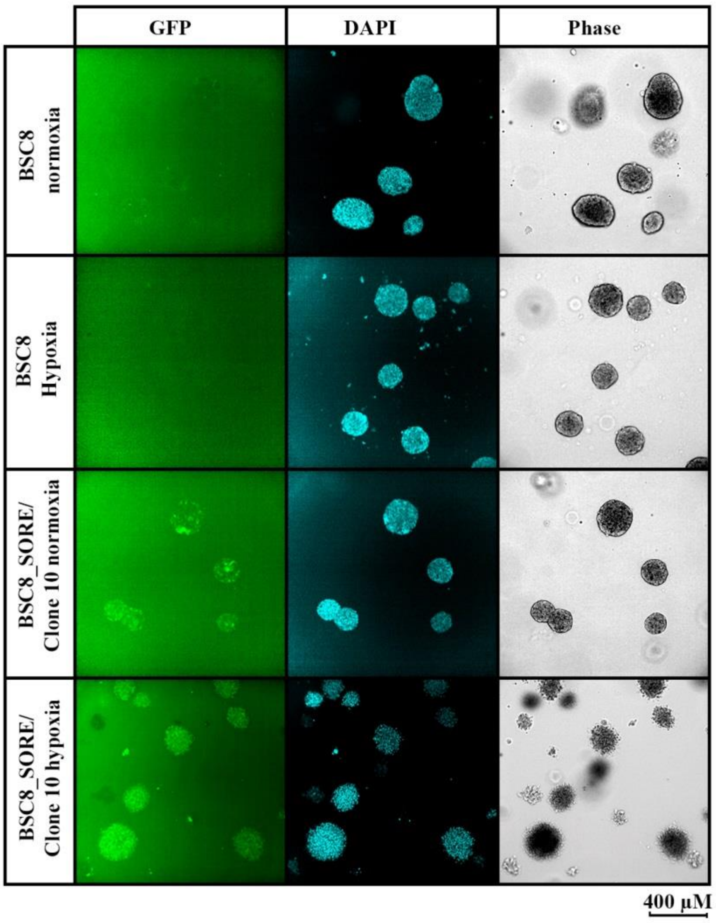

Suppl. Fig. S3. The clonogenic activity of BSC8 tumor cells. Growth of cell clones of cell lines BSC8 and BSC8_SORE+ in semi-liquid agar. Identical sizes and shapes of the formed colonospheres are noted. On the section of colonies of BSC8_ SORE+, the presence of green fluorescent cells is noted. There is a visible increase in the number of these cells during cultivation of the colonospheres under hypoxic conditions. Colonospheres formed in hypoxia are looser in structure. x20.

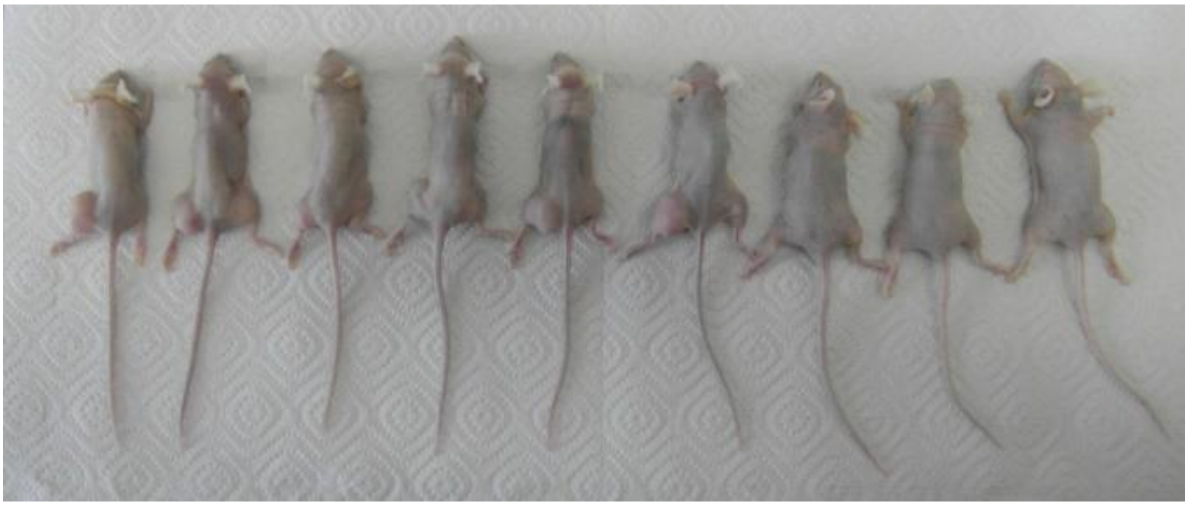

Suppl. Figure S4. Comparison of the tumorogenic activity of the "stem-like" BSC8_SORE+ cells and original BSC8 cells. , the sizes of tumors/ measured in mice 40 days after the injection of BSC8_SORE+ cells (left thigh) and BSC8 (right thigh) are shown. Evaluation of the significance of the differences using the Student's t-test showed that the sizes of tumors generated by BSC8 and BSC8_SORE+ cells significantly different on day 40, $p < 0.01$.

A

PIN OF THE UP-REGULATED GENES IN **BSK8SORE+ cells**
VS CONTROL BSK8 CELL LINE

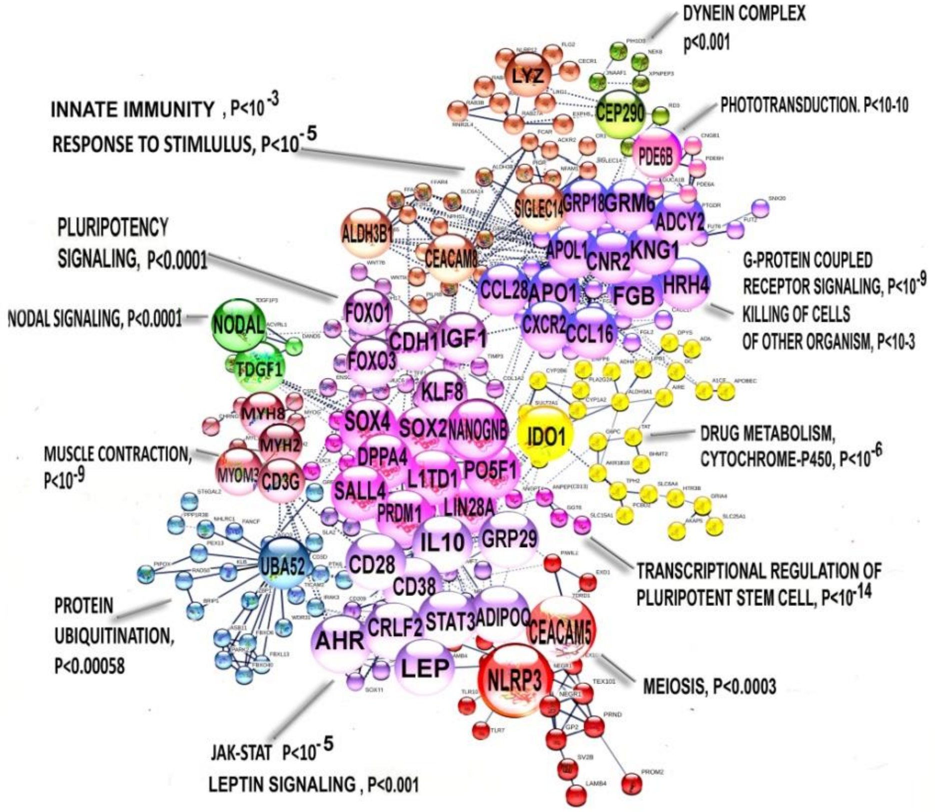

B

PIN OF THE DOWN-REGULATED GENES IN **BSK8SORE+ cells**
VS CONTROL BSK8 CELL LINE

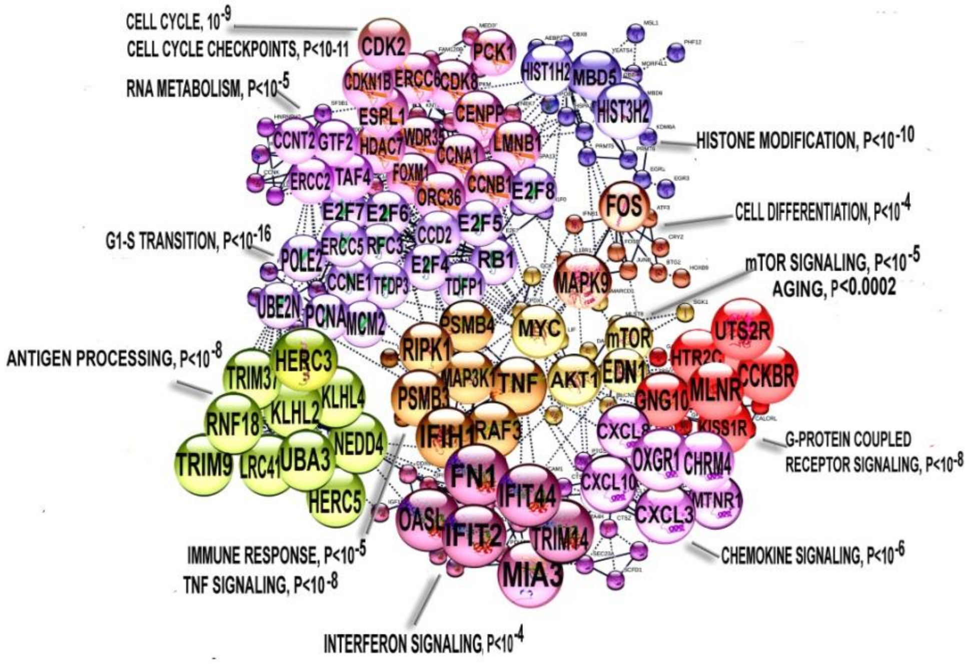

Suppl. Figure S5 (The extended legend for Fig.7 form the main text). The most connected components of protein interaction networks of the up- and down-regulated genes in the stem cell enriched sub-population of the CSC-like cell line vs original population of the BSC8 cell line. **(A)** The PPI network for the up-regulated genes. The most important clusters are involved in the regulation of pluripotency and development, drug resistance and response to other organisms, nodal signaling and male gametogenesis. Three clusters of pluripotency and development (hubs are SOX2, SOX4, SALL4, PO5F1, NANOGNB and LIN28A; hubs are FOXO3, KLF8, IFG1 and hubs STAT3, LEP, CD28, CD38, CRLF2, ARH), a cluster of nodal signaling hubbed by NODAL and TDGF1 and a cluster of gametogenesis hubbed by NLRP3 and CEACAM5 uncover clear induction of stemness. A cluster of genes related to drug metabolism through cytochrome pathway (IDO1 is a hub gene) confirms increased drug resistance. Overall, the induction of these clusters suggest that CSC-like cell line shows less mature phenotype with features of drug resistance compared to the original BSC8 cell population. (**B**) PPI network for the down-regulated genes. The main clusters are presented by genes regulating cell cycle and G1-S transition, differentiation, aging and inflammation. Two clusters implicated in cell - cycle regulation (the main hubs are E2F4-8, CCNE, POLE, PCNA and RB1 and hubs CCNA, CCNB1, CDKN1B, CENPP, CDK2, 8, FOXM1 and other genes) point to decreased mitotic activity. Clusters of cell differentiation (hubs MAPK9 and FOS) and aging (hubs mTOR, AKT1, MYC and EDN1) indicate younger and less differentiated molecular phenotype. Inflammation related clusters, including clusters of interferon signaling (hubs IFIT2, IFIT44, TRIM14 and others), TNF signaling (hubs TNF, RIPK1, MAP3K1, IFIH1, TRAF3 and others) and chemokine signaling (hubs CXCL3, 8, 10, MTNR1, CHMR4 and OXGR1) suggest depressed inflammatory response. Altogether, these changes complement the picture provided by the induced network and suggest that CSC-like cell line reveals features of retarded proliferation, immune suppression and rejuvenation compared to the original BSC8 cell population. The network was constructed using STRING server at interaction confidence > 0.9. Clusterization was done with K-means clustering. Hub proteins, that is, the ones having more than 5 connections, are marked with multicolor large buttons; node proteins are indicated with plain small buttons. Fold expression difference is not less than 10 folds.
