# Supplementary material for: Isolation and Characterization of Human Colon Adenocarcinoma Stem-Like Cells Based on the Endogenous Expression of the Stem Markers
Source: Int J Mol Sci. 2021 Apr 28;22(9):4682. doi: 10.3390/ijms22094682 (PMC8124683; doi:10.3390/ijms22094682)
Supplement: Supplementary file 1 [file ijms-22-04682-s001.zip › Supplementary methods.pdf]

Supplemental methods - The extended version of RNA seq - and bioinformatic analysis methods

Transcriptome sequencing and analysis (Section 11 in the main text)

NGS was done on the Illumina platform by parallel measurement of three biological samples both for BSC8_SORE+ and control cells with read length 150 nt. The processing of NGS reads was done similarly to the previous work [1]. The reads were trimmed using Trimmomatic software with default parameters [2]. The trimmed reads were mapped to canonical nonredundant human transcriptome presented in RefSeq database[3] using Bowtie 2 software[4]. This aligner became a de facto standard within mapping pipelines and shows a remarkable tolerance both to sequencing errors and indels [5]. Bowtie 2 was used with 'very-sensitive' preset of parameters, which allows the most sensitive and accurate mapping (at the expense of speed). Only the nonambiguous mappings were counted.

The obtained gene counts were analyzed using the 'limma' package (implemented in R environment) specially developed for whole transcriptome analyses of differentially expressed genes [6]. Comparison of different software packages showed that limma is the method of choice for our goals [7]. After recommendation in limma manual, genes with counts below 10 in all probes were discarded. This procedure gave 18,810 genes for analysis of differential gene expression. All data normalization methods presented in limma (quantile, scale) were tested as well as the trimmed mean method from the edgeR package [8]. The results were similar. The results obtained with quantile normalization are shown.

Gene module analysis (Section 12 in the main text)

The gene module enrichment analysis was done similarly to previous work [9]. The biological processes were taken from GO database [10]. For each GO category (process), all its subcategories were collected using GO acyclic directed graphs, and a gene was regarded as belonging to a given category if it was mapped to any of its subcategories. As a source of molecular pathways, the NCBI BioSystems was used [11]. The redundancy of this resource, which is a most complete compendium of molecular pathways from different databases, was removed by uniting entries with identical gene sets.

The contrast test was used for analysis of difference in gene expression folds. In this test the mean expression fold of genes belonging to each process/pathway is compared with the mean fold of total gene set. For evaluation of two-tailed statistical significance of an obtained contrast between these folds, 20,000 random samplings were taken from total gene set (of a size equal to the number of genes in a process/pathway). This method is preferable to

parametric or non-parametric tests because normal distribution that is required for parametric tests is usually absent, whereas non-parametric tests can lose a considerable amount of information. The random-sampling test is distribution-independent and retains all in-formation. The adjustment of obtained p-values for multiple comparisons was done after Storey and Tib-shirani [12]. This procedure gives q-value, which can be considered as p-value corrected for multiple tests.

Protein-protein interaction network analysis (Section 13 in the main text)

The protein-protein interactions (PPI) were taken from the STRING database [13]. We choose it because STRING places its focus on functional relationship between two proteins, contributing to a common biological purpose and contains interactions from multiple sources: experimental interactions imported from primary databases, pathways from manually curated databases and statistical and se-mantic links between proteins, obtained from Medline abstracts and a large collection of articles. STRING is claimed "to collect, score and integrate all publicly available sources of protein–protein inter-action information" [13]**.** The PPI networks (PINs) were visualized using STRING server. We analyzed dense connected components of PINs for proteins encoded by genes differing in expression between BSC8_SORE+ cells and the control BSC8 cell line by more than tenfold. The up-regulated and down-regulated genes were analyzed separately. PINs consist of nodes and edges, where each node stands for a protein and the edges represent interactions [14]. The number of edges per a node characterizing the number of interacting proteins is termed a degree. Nodes with highest degree are defined as hubs[15]. The degree is a fundamental parameter that is usually adopted to evaluate the nodes in a network for the identification of evolutionary conserved causal regulators in modular organization of networks [16-18]

References:

1. Vinogradov AE, Shilina MA, Anatskaya O V., Alekseenko LL, Fridlyanskaya II, Krasnenko A, Kim A, Korostin D, Ilynsky V, Elmuratov A, Tsyganov O, Grinchuk TM, et al. Molecular genetic analysis of human endometrial mesenchymal stem cells that survived sublethal heat shock. *Stem Cells Int* 2017;2017.
2. Bolger AM, Lohse M, Usadel B. Trimmomatic: A flexible trimmer for Illumina sequence data. *Bioinformatics* 2014;30:2114–20.
3. Agarwala R, Barrett T, Beck J, Benson DA, Bollin C, Bolton E, Bourexis D, Brister JR, Bryant SH, Canese K, Cavanaugh M, Charowhas C, et al. Database resources of the National Center for Biotechnology Information. *Nucleic Acids Res* 2018;46:D8–13.
4. Langmead B, Salzberg SL. Fast gapped-read alignment with Bowtie 2. *Nat Methods* 2012;9:357–9.

5. Lindner R, Friedel CC. A Comprehensive Evaluation of Alignment Algorithms in the Context of RNA-Seq. *PLoS One* 2012;7.

6. Ritchie ME, Phipson B, Wu D, Hu Y, Law CW, Shi W, Smyth GK. Limma powers differential expression analyses for RNA-sequencing and microarray studies. *Nucleic Acids Res* 2015;43:e47.

7. Seyednasrollah F, Laiho A, Elo LL. Comparison of software packages for detecting differential expression in RNA-seq studies. *Brief Bioinform* 2013;16:59–70.

8. Zhou X, Lindsay H, Robinson MD. Robustly detecting differential expression in RNA sequencing data using observation weights. *Nucleic Acids Res* 2014;42.

9. Vinogradov AE, Anatskaya O V. Gene Golden Age paradox and its partial solution. *Genomics* 2019;111:115–26.

10. Carbon S, Douglass E, Dunn N, Good B, Harris NL, Lewis SE, Mungall CJ, Basu S, Chisholm RL, Dodson RJ, Hartline E, Fey P, et al. The Gene Ontology Resource: 20 years and still GOing strong. *Nucleic Acids Res* 2019;47:D330–8.

11. Storey JD, Tibshirani R. Statistical significance for genomewide studies. *Proc Natl Acad Sci U S A* 2003;100:9440–5.

12. Hu JX, Thomas CE, Brunak S. Network biology concepts in complex disease comorbidities. *Nat. Rev. Genet.*2016;17:615–29.

13. Chen C, Shen H, Zhang LG, Liu J, Cao XG, Yao AL, Kang SS, Gao WX, Han H, Cao FH, Li ZG. Construction and analysis of protein-protein interaction networks based on proteomics data of prostate cancer. *Int J Mol Med* 2016;37:1576–86.

14. Vallabhajosyula RR, Chakravarti D, Lutfeali S, Ray A, Raval A. Identifying Hubs in protein interaction networks. *PLoS One* 2009;4.

15. Zhang B, Gaiteri C, Bodea LG, Wang Z, McElwee J, Podtelezhnikov AA, Zhang C, Xie T, Tran L, Dobrin R, Fluder E, Clurman B, et al. Integrated systems approach identifies genetic nodes and networks in late-onset Alzheimer's disease. *Cell* 2013;153:707–20.
